# Seasonal variability in community structure and metabolism of active deep-sea microorganisms

**DOI:** 10.1093/ismejo/wraf214

**Published:** 2025-09-23

**Authors:** Yinghui He, Federico Baltar, Yong Wang

**Affiliations:** Institute for Ocean Engineering, Shenzhen International Graduate School, Tsinghua University, Shenzhen, Guangdong, 518055, P.R. of China; College of Oceanography and Ecological Science, Shanghai Ocean University, Shanghai, 201306, P.R. of China; Department of Functional and Evolutionary Ecology, University of Vienna, Vienna, 1010, Austria; Institute for Ocean Engineering, Shenzhen International Graduate School, Tsinghua University, Shenzhen, Guangdong, 518055, P.R. of China; Shenzhen Key Laboratory of Advanced Technology for Marine Ecology, Shenzhen International Graduate School, Tsinghua University, Shenzhen, Guangdong, 518055, P.R. of China

**Keywords:** microbial community, transcriptomics, carbon cycle, MISNAC

## Abstract

Learning about the metabolic activities and adaptations of deep-sea microbes is challenging, as sample collection and retrieval often cause RNA degradation and microbial community shifts. Here, we employed an *in situ* DNA/RNA co-extraction device to collect 18 time-series nucleic acid samples during winter and summer in the South China Sea, minimizing sampling perturbation for metatranscriptome and metagenome analyses. Between the two seasons, the prokaryotic microbiota showed seasonal variations in species composition. *Burkholderiales* dominated in summer, whereas *Pseudomonadales*, *Bacillales*, and *Rhodobacterales* were enriched in winter. However, the dominant transcriptionally active taxa affiliated with *Nitrososphaerales*, MGIII, SAR324, UBA11654, *Marinisomatales*, and *Poseidoniales* remained largely stable across seasons. Among eukaryotes, *Ciliophora* were the most active, whereas *Retaria* were abundant but inactive. Despite the stable active prokaryotic community, metabolic profiles differed significantly between seasons. In the winter, autotrophic microorganisms, particularly *Nitrososphaerales*, exhibited higher CO_2_ fixation activity via the 3HP/4HB cycle, accompanied by enhanced ammonia oxidation for energy generation. In addition, CO oxidation activity was also elevated. In the summer, the primary source of energy originated from heterotrophic microorganisms capable of utilizing fatty acids, benzoate, and H_2_, likely relying on anaerobic respiration within organic particles. This may relate to nutrient source variations as reflected by the different levels of microbial network complexity between the two seasons. Altogether, our *in situ* metatranscriptomes revealed the metabolic activities and adaptations of active microbial groups across seasons, providing a basis for identifying the microbial contributors to elemental cycles in the deep ocean.

## Introduction

Marine microbes from the surface and deep ocean are key players regulating the carbon cycle in the ocean [1, 2]. The microbial communities and metabolic activities are rather dynamic in different locations and depths. In the deep sea, the microbial community is usually composed of indigenous inhabitants and some microbes associated with descending organic particles [3–5]. Considering that the deep-sea organisms are to some extent dependent on photosynthesis of the surface ocean, the deep ecosystem is thus under dramatic impacts by the primary production of the surface ocean [6]. Seasonal variation of the surface ecosystem is a common re-occurring phenomenon [7]. For instance, the northwestern part of the South China Sea (SCS) in summer is characterized by nutrient-enriched waters, low dissolved oxygen, elevated chlorophyll a concentrations, and enhanced primary production, which may drive seasonal variations in surface microbial communities [8, 9]. The varying downward fluxes of ocean surface productivity might impact the composition and metabolic profile of deep-sea microbial communities [9–11], although only ~1% of the organic carbon can reach 3000 m depth [12]. In deep waters (890 m depth) off the coast of Southern California, the entire microbial community was reported to undergo seasonal changes [10]. Similarly, in the South Pacific Ocean, a seasonal linkage between surface and deep (500 m) prokaryotic community composition has been observed, driven not only by summer particle sinking but also by winter water column overturning [11]. However, the seasonal dynamics of fundamental metabolic processes within the active deep-sea microbiota remain poorly understood. To uncover the active microbiota and their microbial metabolisms in situ, integrating metagenomics with metatranscriptomics datasets from the deep ocean is essential. Devices designated for microbial genomic and transcriptomic *in situ* extraction have been recently developed. For example, the environmental sample processor (ESP) platform with a function of autonomous extraction of marine microbial DNA/RNA was used to analyze in situ microbial samples for metatranscriptomics data in marine surface waters [13, 14]. In addition, the 3G ESP (third-generation Deep Sea Environmental Sample Processor) [15] is specifically designed to fit on the AUV (autonomous underwater vehicle). It can collect samples in various depths along the water column [15]. Another variant, the Deep-sea ESP (D-ESP), has been developed for similar studies at depths of up to 4000 m [16]. These advanced sampling technologies enable critical research into deep-sea microbial ecosystems, yet reports focusing on the study of *in situ* variations in gene expression of deep-sea microorganisms are extremely limited. Studying the temporal variations of the gene expression of deep-sea microorganisms can help us gain a better understanding of deep-sea ecosystems, thereby expanding our knowledge of their fitness and ecological role in this wide and underexplored ecosystem.

To investigate seasonal variation in deep-sea microbial activity (eukaryotic and prokaryotic), we applied the Multiple In Situ Nucleic Acid Collections (MISNAC) method [17] at depths of 1022–1084 m in the South China Sea. MISNAC enables time-course *in situ* nucleic acid collection, minimizing recovery-induced biases [17, 18]. Here, we integrated metagenomic and metatranscriptomic data to compare microbial community composition and gene expression across winter and summer, aiming to identify the active taxa and their ecological strategies under different seasonal regimes. In contrast to seasonal differences in taxonomic composition, transcriptomic profiles revealed a consistent group of active deep-sea prokaryotes across both seasons. Nonetheless, the microbial taxa employed distinct ecological strategies for organic carbon and energy acquisition. Understanding such metabolic shifts provides insight into how microbial populations adapt and contribute to elemental cycling in seasonally dynamic deep-sea ecosystems.

## Materials and methods

### *In situ* collection of deep-sea microbial samples using Multiple In Situ Nucleic Acid Collections

The samples were collected at 1022–1084 m depths in the SCS using the Phoenix lander equipped with the MISNAC [17] (Fig. S13). Collection of the winter samples was conducted by six working units of MISNAC through two separate deployments of the lander (FH38 and FH39). Both deployments were conducted within a continuous 24-h period between 23 and 25 November (winter) 2019 (Table S1). The summer samples were collected between 9 and 11 June 2020 (deployments FH41 and FH42 cruises) using the same method. All the sampling sites were close to each other and were located adjacently on a wide flat seafloor. The MISNAC sampling was performed in three (T2: 15:00–19:00; T3: 19:00–23:00; T4: 23:00–3:00) and six (T1: 11:00–15:00; T2: 15:00–19:00; T3: 19:00–23:00; T4: 23:00–3:00; T5: 3:00–7:00; T6: 7:00–11:00) time intervals with two replicates (D1 and D2) over 2 days in the winter and summer cruises, respectively. Due to cruise arrangements, MISNAC collected the samples at three time intervals (T2–T4) in the summer. Before the MISNAC deployment, 3 l of 70% alcohol and deionized distilled water were flushed separately into the hose, filter chamber, and valve box of MISNAC. The water was then filtered with a 0.22 μm polycarbonate membrane (Millipore, Bedford, MA, USA) and served as a control to deplete contaminants in subsequent omics data. About 30 min after the lander reached the seafloor, the MISNAC device [17] automatically commenced its operations to avoid absorption of floating sediment particles disturbed by the lander. Water prefiltered through a 1 mm polycarbonate membrane was pumped into the filtration chamber of MISNAC subunits and filtered through 0.22 μm polycarbonate membrane with 142 mm diameter (Millipore, Bedford, MA, USA). Cell lysis buffer (50 mM Tris-HCl, 150 mM NaCl, 1 mM EDTA, 10% GuHCl, 10% DTT, 1% Triton X-100, 0.2% Proteinase K, and 0.1% lysozyme) was injected into the filtration chamber and maintained for 30 min to lyse the microbial cells on the filter membrane. The lysate of the microorganism flowed out of the filtration chamber and was mixed with an equal volume of 90% ethanol. The precipitated nucleic acids were absorbed by polymer columns (Tiangen, Beijing, China; #RK130-C). Each of the working units filtered ~80 l of water for *in situ* cell lysis and nucleic acid collection by polymer columns. The nucleic acids were washed off by distilled deionized water with 4000 rpm centrifugation onboard. The quality and quantity of nucleic acids were estimated by the Qubit DNA and RNA Assay Kit by a Qubit 2.0 fluorometer (Thermofisher, Waltham, MA, USA) and were transferred to a −20°C refrigerator for subsequent experiments. The Phoenix lander was equipped with a CO_2_ sensor (Pro-Oceanus, Bridgewater, NS, Canada) and a Conductivity-Temperature-and-Depth (CTD) sensor (Sea-Bird, Bellevue, WA, USA) to monitor the environmental factors during the MISNAC sampling. The concentrations of phosphorus, silicon, NO_2_^−^, NH_4_^+^, and NO_3_^−^ were measured using the winter water samples obtained by a Niskin bottle.

### Metatranscriptomic and metagenomic library preparations

From each MISNAC column, we obtained >1 μg nucleic acids. For DNA library preparation, 18 MISNAC samples and the blank control were incubated with 0.1 ng/μl RNaseA (TaKaRa, Dalian, China) at 37°C for 10 min to remove RNA fragments. A total of 100 ng DNA (1 ng DNA for the control) was fragmented to ~350 bp by ultrasound with Covaris M220 Focused ultrasonicator (Covaris, Massachusetts, USA), and then, the high-throughput metagenome libraries were constructed by using VAHTS Universal DNA library prep kit for Illumina V3 (Number: N411-01/02/03, Vazyme, Nanjing, China) according to the manufacturer’s protocol. For RNA library preparation, DNA fragments were removed from 18 MISNAC nucleic acid samples using the Turbo DNA-free Kit (Ambion, Carlsbad, CA, USA) according to the instructions. In order to ensure the removal of DNA in nucleic acid samples, the universal primers 341F (5′- CCTAYGGGRBGCASCAG-3′) and 802R (5′- TACNVGGGTATCTAATCC-3′) [19] of the V3–V4 variable region of 16S rRNA gene were used for PCR amplification. Double-stranded cDNA was synthesized from 10 ng of RNA using the Ovation RNA-Seq System V2 Kit (Qiagen, Hilden, Germany) according to the manufacturer’s instructions. The VAHTS universal V3 DNA library prep kit (Vazyme, Nanjing, China) for Illumina was used to construct a high-throughput library of metatranscriptome. The high-throughput libraries of the control were also constructed according to the above-mentioned methods. The libraries were sequenced on a NovaSeq 6000 System (Illumina, San Diego, USA) with PE 2 × 150 bp.

### Sequencing and processing of raw Illumina sequencing data

All analyses were performed on the local server, except where explicitly stated otherwise. Raw reads were trimmed to remove adapters and then filtered using fastp (v.0.21.0) with parameters (-f 1 -t 0 -F 1 -T 0 -w 24 -c -q 20 -u 20 -g -W 5 -3 -l 50) [20]. Low-quality reads (assigned by a quality score < 20 for >20% of the read length), unpaired reads, and those <50 bp were removed. FastUniq [21] was used to remove duplicated paired short reads with default parameters. Metagenomic reads were mapped against the control sequencing data and a contaminant database containing mouse and human genomes, as well as genomes of common laboratory contaminants downloaded from NCBI [22], using Bowtie2 [23] with default parameters to filter out contaminant reads. For metatranscriptomes, rRNA reads (5S, 5.8S, 16S, 18S, 23S, and 28S) were removed using SortMeRNA [24] software with an e-value cutoff of 1E-5.

### Taxonomic classification and biodiversity estimates using 16S and 18S rRNA gene sequences

The 16S and 18S rRNA fragments longer than 100 bp were identified from qualified reads in winter and summer metagenomic and metatranscriptomic samples by utilizing rRNA_HMM [25]. The fragments that mapped to V4 regions of 16S rRNA genes and V9 regions of 18S rRNA were further identified using hmmsearch [26]. The extracted reads for V4 regions of 16S rRNA genes and V9 regions of 18S rRNA were imported into QIIME2 (v.2022.2) [27] for analysis of biodiversity and taxonomic classification. The sequences were dereplicated using the dereplicate-sequences function. Amplicon samples (SCS15 and SCS17) of surface water from the sites near the sampling locations were downloaded from the NCBI Sequence Read Archive under the accession numbers SRR1292422 and SRR1292423 [28].

The reads were clustered to OTUs with a similarity level of 97%. The longest read from each OTU was selected as the representative read for taxonomic classification using the QIIME2 classify-sklearn plugin against the SILVA 138 database [29]. Mitochondrial and chloroplast OTUs were removed. The relative abundances of the taxa, based on OTU classification, were used for PCoA. Differences in taxonomic profiles between groups were evaluated using Statistical Analysis of Metagenomic Profiles (STAMP) [30] software v2.1.3 with Welch’s *t*-test.

### Metagenome assembly and genome binning.

Two methods were employed to perform the metagenomic assemblies. Each of the 18 samples was assembled individually using metaSPAdes [31] (v3.15.2) software with the parameters “-min-contig-len 1300 -k 21,33,55,77,99,127.” In addition, all reads from each season (winter or summer) were co-assembled with MEGAHIT [32] (v1.2.8) using a kmer range of 21–141 and k-step of 10 (−-min-contig-len 300 -m 0.9 --k-min 21 --k-max 141 --k-step 10). The metagenome-assembled genomes (MAGs) were binned using the MaxBin, MetaBAT, and CONCOCT software integrated by MetaWRAP [33] software (v1.2.1), based on the two sets of assemblies. The bin refinement module was performed with default parameters. The MAGs with completeness >50% and contamination <10% (metawrap bin_refinement “-c 50 -x 10”) were identified and selected by CheckM [34]. This yielded a total of 1175 MAGs. De-redundancy of the MAGs was processed using the dRep [35] software with an average nucleotide identity (ANI) threshold of 95% at the species level (−pa 0.95 -sa 0.99 -nc 0.30 -cm larger -p 40 -comp 50 -con 10), which resulted in 423 prokaryotic MAGs.

### Phylogenomics analysis

To classify the obtained nonredundant MAGs, we used the GTDB-tk [36] software (v2.1.0) integrated with database r207. A total of 423 MAGs from this study and the closest reference genomes recruited from the Genome Taxonomy Database (GTDB) database [36] were used to construct a phylogenomic tree based on the concatenated alignment of 43 commonly conserved proteins predicted by CheckM [34]. We aligned the protein sequences of the marker genes using Mafft (v7.515) [37] with the setting --maxiterate 1000 --localpair, and then removed the poorly aligned regions in each aligned conserved protein using trimAl (v.1.4) [38]. The maximum-likelihood algorithm was used to construct the tree using IQ-TREE (version 2.2.0) [39] with the model “MFP” (-m MFP -B 1000 -alrt 1000). The phylogenetic trees were visualized using iTOL v.7 (https://itol.embl.de/).

The relative abundance of each MAG in the metagenomes and metatranscriptomes was estimated with CoverM v0.6.1 (https://github.com/wwood/CoverM) in genome mode. For metagenomes, we used the parameters “--min-read-aligned-length 50 --min-read-percent-identity 0.95 --min-covered-fraction 0.1 --min-read-aligned-percent 0.75.” For metatranscriptomes, we used “--min-read-aligned-length 50 --min-read-percent-identity 0.95 --min-covered-fraction 0.01.”

### Functional annotation, metatranscriptomics, and metatranscriptomics analysis

Open reading frames (ORFs) and translated protein sequences for individual MAGs were predicted using prodigal (v.2.6.3) with option “-p meta.” KEGG Orthologs (KOs) [40] were assigned to these protein sequences via KofamScan [41] (v.1.1.0) and GhostKOALA (v.2.2) [41]. For genes that could not be distinguished by KofamScan and GhostKOALA, such as *amoA*, *pmoA*, and NiFe hydrogenase genes, we identified these functional genes in the MAGs using DIAMOND v2.1.9 (--max-target-seqs 1, --max-hsps 1) by searching their protein sequences against a custom database of metabolic marker proteins from the Green Lab (10.26180/c.5230745) [42, 43]. To enhance this database, we supplemented it with *amoB* and *pmoB* sequences retrieved from NCBI and UniProt. To summarize the frequency of the functional genes across different phyla, the occurrence frequency of each marker gene was calculated by counting its presence in the MAGs within each phylum. The heatmap was generated using R to visualize the results. For transcriptional analysis of the above functional genes, the relative abundance (TPM value) of their ORFs in each of the non-rRNA normalized metatranscriptomic datasets was calculated using CoverM v0.6.1 (https://github.com/wwood/CoverM) in contig mode (with the following settings: “-m tpm --min-read-aligned-percent 0.75 --min-read-percent-identity 0.95 --min-covered-fraction 0” in contig mode). To normalize the TPM values calculated using the different metatranscriptomics data, we divided the TPM values by the summed counts of 40 universally conserved, single-copy, and constitutively expressed marker genes suggested by mOTUs1 [44, 45] (K06942, K01889, K01887, K02950, K02992, K02967, K02867, K02863, K03043, K02926, K02906, K02886, K02890, K02982, K02874, K02931, K02994, K02933, K02988, K02952, K02948, K02871, K02996, K01892, K01875, K02956, K02965, K02961, K02878, K02876, K03076, K03040, K01883, K02881, K01869, K02986, K01873, K01409, K03106, K03110) (Table S7). These single-copy phylogenetic marker genes were thought to be particularly suitable for normalization of gene transcriptional level to approximately per-cell gene transcript copies. TPM values of ORFs in the metagenomes were calculated by CoverM software with the setting “-m tpm --min-read-aligned-percent 0.75 --min-read-percent-identity 0.95 --min-covered-fraction 0” in contig mode. Normalization of the TPM values obtained using different metagenomic datasets was conducted using the total counts of 10 single-copy house-keeping genes (K06942, K01889, K01887, K01875, K01883, K01869, K01873, K01409, K03106, K03110) as suggested by mOTUs2 (Table S8) [44, 45].

### Co-occurrence networks and keystone taxa identification

Co-occurrence networks were constructed based on the 16S rRNA gene sequences extracted from metatranscriptomic datasets, which were taxonomically classified and clustered into OTUs using QIIME2. The OTU abundance profiles across different samples were then used for calculating Spearman’s correlation [46]. In the networks, OTUs represent nodes, and correlations between OTUs are displayed as edges. Benjamini and Hochberg false discovery rates (FDRs) were used to adjust the *P*-values (*P* ≤ .05) in the correlation [46]. The network properties were obtained by using the igraph package [46]. A co-occurrence event was considered a robust correlation if Spearman’s correlation coefficient (*r*) was >0.8 and statistically significant (*P* < .05). Networks were explored and visualized with the interactive platform gephi [47], and network topological parameters including node and edge numbers, average degree, modularity, average clustering coefficient, and average path length were calculated by gephi [47].

## Results

### Microbial community composition varied with season and time

**Figure 1:**
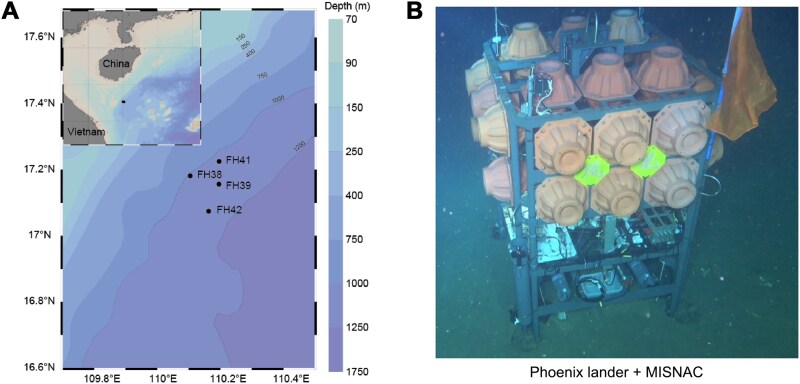
Sampling sites and sampling device. (A) The sampling sites of the summer and winter cruises at a depth between 1022 and 1084 m. (B) Sampling device MISNAC equipped on a deep-sea lander. The map of sampling sites was generated using Data Ocean View software.

In two research cruises conducted in June 2020 (summer) and November 2019 (winter), ~80 l of deep-sea water was filtered for *in situ* DNA/RNA co-extractions with each MISNAC working unit at four adjacent sites between 1022 and 1084 m depth located on the same seafloor plain in the northwestern SCS (Fig. 1A and B, Table S1). Environmental factors in the winter cruises FH38 and FH39 exhibited constant salinity (average value was 34.28 ± 0.01 PSU) and temperature (the average value was 4.37 ± 0.04°C) during the sampling. Similarly, the summer cruises FH41 and FH42 also observed stable salinity (with an average value of 34.21 ± 0.01 PSU) and temperature (with an average value of 4.16 ± 0.04°C). Dissolved CO_2_ ranged between 1000 and 1200 ppm in the summer cruise. In the winter cruise, dissolved CO_2_ ranged between 970 and 1100 ppm. The concentrations of phosphorus, silicon, NO_2_^−^, NH_4_^+^, and NO_3_^−^ in the water samples showed lower NH_4_^+^ and NO_3_^−^ in summer compared to winter (Table S2).

**Figure 2:**
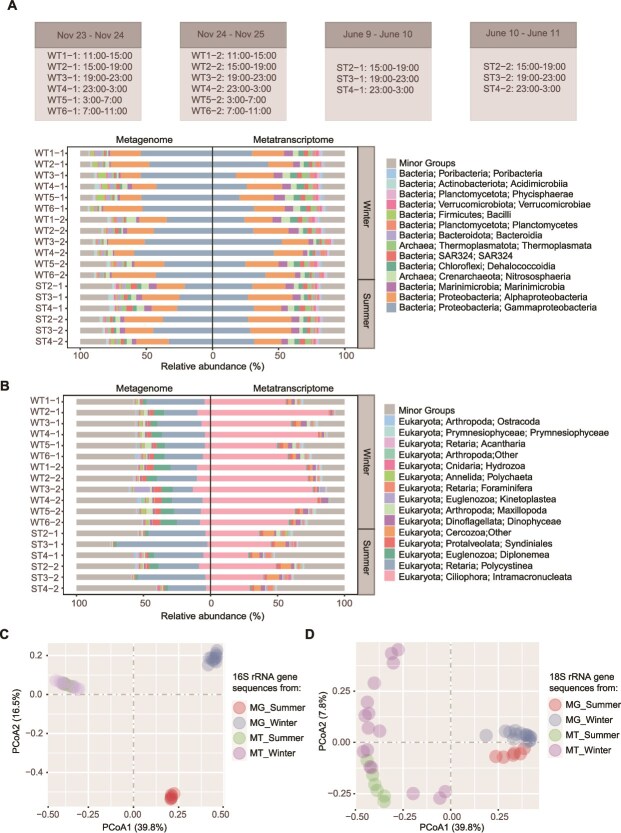
Seasonal variation of the community structures at the sampling sites. Taxonomic composition of (A) prokaryotic and (B) eukaryotic communities at the class level based on the classification of 16S and 18S rRNA gene sequences. The principal coordinate analysis (PCoA) plots for prokaryotic (C) and eukaryotic (D) community structures were based on Bray–Curtis dissimilarity matrices calculated using species-level taxonomic classification of 16S and 18S rRNA gene sequences, respectively. WT, winter; ST, summer; MG, metagenomic reads; MT, metatranscriptomic reads. Sample IDs are listed in Table S1.

The collected nucleic acid yielded 333 and 168 Gbp of Illumina raw data for 18 metagenomes and 18 metatranscriptomes. (Table S3). After quality control and decontamination, 16S and 18S rRNA gene fragments in the Illumina reads for the V4 and V9 regions were extracted for taxonomic classification and biodiversity estimation. About 6.2% and 6.6% of the 16S rRNA gene sequences in the summer and winter metagenomes were assigned to previously unclassified species. On average, 9.1% of the 16S rRNA gene sequences of the winter metatranscriptomes were <97% similar to SILVA 138 entries, indicating new species-level taxa. The most abundant OTUs (97% similarity, based on 16S rRNA gene sequences) in all metagenomes were affiliated with *Gammaproteobacteria* (41.8%), *Alphaproteobacteria* (23.1%), and *Marinimicrobia* (8.8%) (Figs 2A, S1, and S2). They were also most abundant in the metatranscriptomes and accounted for 32.3%, 23.7%, and 6.1% of the 16S rRNA gene sequences extracted from the metatranscriptomes (Figs 2A, S1, and  S2). At lower taxonomic levels, *Rhodospirillales* displayed significantly higher transcriptional abundance in the summer metatranscriptomes (Welch’s *t*-test; *P* ≤ .05), whereas *Pseudomonadales*, *Methylococcales,* and SAR202 dominated the winter metatranscriptomes (Fig. S2). To investigate the connectivity between the deep-sea and surface microbial communities, the relative abundance of surface prokaryotes was obtained using 16S rRNA gene sequences extracted from public metagenomes for two water samples near the sampling sites [28]. The results showed that three prokaryotic species affiliated with *Rhodobacteraceae* and SAR11 clades I and II (*Proteobacteria*) accounted for at least over 1% of the communities in both the deep-sea MISNAC and surface samples (Table S4). The most dominant species in the surface samples consists of *Prochlorococcus* MIT9313, *Candidatus* Actinomarina, and SAR11 clade Ia (on average, 21.4%, 9.9%, and 9.6% of the communities).

Eukaryotic microbial communities based on 18S rRNA gene sequences of the metagenomes were dominated by *Polycystinea* (31.7%), *Diplonemea* (6.6%), and I*ntramacronucleata* (6.5%) (phylum: *Retaria*, *Ciliophora,* and *Euglenozoa*), whereas those based on the metatranscriptomes were overwhelmingly represented by *Ciliophora* (57.9%) and *Cercozoa* (5.1%) (Figs 2B and S3). *Ciliophora* was detected at nearly 10-fold higher relative abundance in the 18S OTUs from the transcriptomes compared with the metagenomes. By contrast, the relative abundance of *Retaria* exhibited a reverse trend to *Ciliophora* (Figs S3 and S4). The *Ciliophora* were composed of *Intramacronucleata* known as common predatory marine ciliates; the *Retaria* were represented by *Polycystine* and *Acantharia*, which belong to radiolarians, probably originated from the surface ocean.

Principal coordinate analysis (PCoA) revealed high dissimilarity in metagenome-based prokaryotic communities between seasons (Figs 2C and S5A). Conversely, the community structures based on the 16S rRNA gene sequences extracted from the metatranscriptomic data were similar for both seasons. We revealed more diversified active eukaryotic microorganisms based on the 18S rRNA gene sequences extracted from the metatranscriptomes, compared with those from the metagenomes, based on the PCoA result (Figs 2D and S5B). This is in a reversal trend compared to the prokaryotic counterparts of the two seasons.

### Genomic consistency and transcriptomic variability of deep-sea microbial in summer and winter

**Figure 3:**
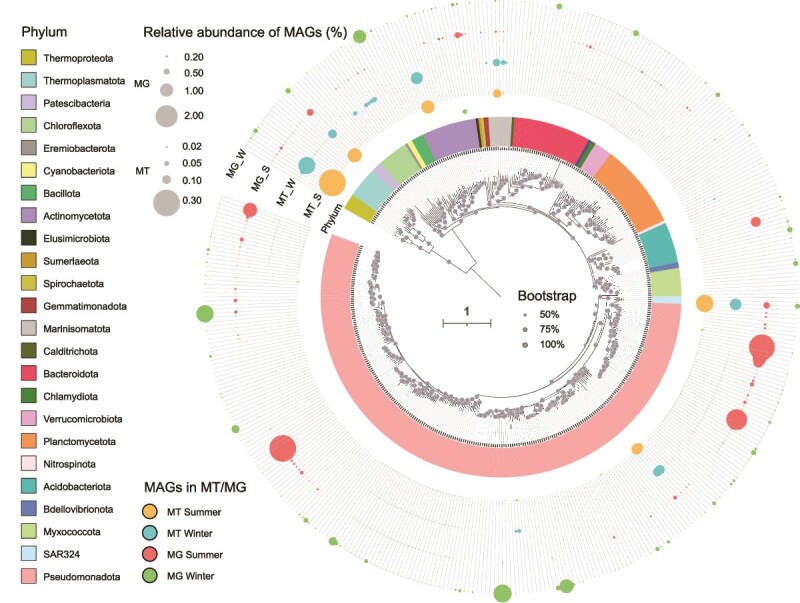
Phylogenomics tree of prokaryotic metagenome-assembled genomes (MAGs) with different relative abundances in seasons. The maximum-likelihood phylogenomics tree was constructed using the conserved proteins encoded by the 423 MAGs representing different species (Table S6). The relative abundance was estimated by the percentage of the metagenomic and metatranscriptomic reads mapped on the MAGs. The scale bar represents one substitution per site on average. The circle size represents the relative abundance of MAGs in different seasons as shown in the metagenomic and metatranscriptomic data, with different circle sizes corresponding to specific abundance percentages. MG, metagenomic reads; MT, metatranscriptomic reads.

The assembly of the 18 metagenomes generated 1175 draft genomes, of which 531 were classified as MAGs with >90% completeness and <5% contamination. The MAG dataset was further dereplicated into 423 MAGs with an ANI of 95% to retain the genomes at the species level (Table S5). On average, 29.5% and 30.1% of the reads in the summer and winter metagenomes were mapped on the contigs from the MAGs (Fig. 3, Table S6).

The classification of the 423 MAGs using the GTDB revealed 18 archaeal and 405 bacterial MAGs representing prokaryotic species affiliated to two archaeal and 24 bacterial phyla. A phylogenomics tree was constructed using the conserved proteins encoded by the 423 MAGs to illustrate their phylogenetic relationships (Fig. 3). The relative abundance of the MAGs in metagenomes was individually calculated (Table S6). The majority of highly active MAGs in both seasons belonged to the same taxonomic groups, including *Thermoproteota*, SAR324, *Pseudomonadota*, *Marinisomatota*, and *Thermoplasmatota*, albeit with different rankings within the top 10 MAGs based on the coverage by metatranscriptomic reads (Figs 3 and S6, Table S6). In reference to metagenomic coverage that differed between the two seasons, the MAGs with the highest relative abundance belonged to the families of *Gammaproteobacteria*, *Alphaproteobacteria*, SAR324, *Thermoproteota*, and *Thermoplasmatota*. Among these, MAGs belonging to NAC60-12, *Nitrosopumilaceae*, and *Thalassarchaeaceae* showed high genomic coverage in both summer and winter metagenomes. However, the MAGs representing *Rhodocyclaceae*, *Caulobacteraceae* and *Burkholderiaceae* (*Aquabacterium*), and *Sphingomonadaceae* (*Novosphingobium*) were highly covered by the summer metagenomes, which were replaced by those for *Sphingomonadaceae* (*Sphingobium*), *Bacillaceae*, *Pseudomonadaceae*, *Moraxellaceae*, *Parvularculaceae*, and *Burkholderiaceae* (*Acidovorax*) in the winter (Figs 3 and S6, Table S6).

### Functional gene profile of the deep-sea microbiomes

All the predicted genes in the 1175 MAGs were further clustered into a nonredundant dataset constituted by 1 569 435 gene clusters with 95% similarity threshold for each cluster. Overall, 190 037 (12.1%) of the clusters were transcribed in the time-course samples as suggested by the mapping rates on the metatranscriptomes. Among the transcribed gene clusters, 118 830 (62.1%) and 176 333 (92.8%) were annotated to a KEGG and an eggNOG orthologous group. The metabolic activities of the microbial lineages were further investigated by assigning metabolic functions to the 715 288 predicted genes in the contigs of the metatranscriptomes. Approximately 40.9 ± 8.0% of the transcribed genes in the MAGs had unknown functions, some of which were among the most actively transcribed in the winter samples (Fig. S7).

Variations in prokaryotic and eukaryotic cell counts across the time-course samples can introduce biases in genomic or transcriptomic analyses. To normalize prokaryotic expression levels and minimize the effect of eukaryotic cell expression in the samples of different seasons, the total expression level of single-copy prokaryotic marker genes, which are typically housekeeping genes expressed consistently across cells, was calculated for the individual metatranscriptomes. The marker genes are fundamental to essential cellular functions, such as ribosomal protein synthesis and cytoskeletal maintenance, which indicates minimal fluctuations in expression level across the samples. Normalizing the metagenomes and metatranscriptomes using the total relative abundance of the marker genes might effectively mitigate discrepancies caused by differences in prokaryotic cell numbers. The normalization was conducted by mOTUs profiler [44, 45, 48–50] (see Materials and Methods) (Fig. S8, Tables S7 and S8).

**Figure 4:**
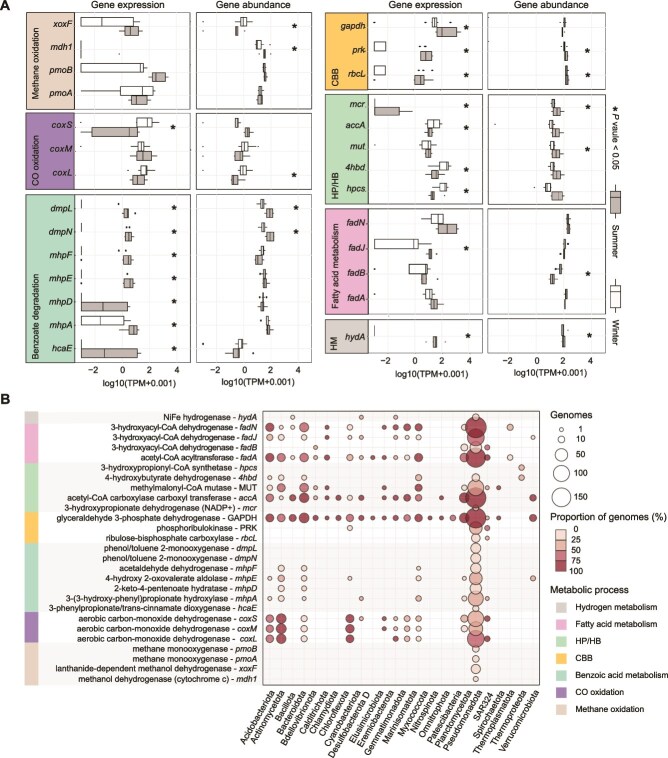
Presence of carbon and hydrogen metabolic genes in different phyla and their relative abundance variations in metagenomic and metatranscriptomic data of the two seasons. (A) Relative abundance of the genes at the genomic and transcriptional levels in the summer and winter samples. All TPM values of the genes mapped by the metagenomic and metatranscriptomic reads have been normalized by mOTUs and transformed using log10(TPM + 0.001). To assess the significance of the differences in TPM values for the same genes between the winter and summer datasets, the Wilcoxon test for independent samples was performed. The genes showing a *P*-value ≤.05 are marked by a star. CBB, Calvin–Benson–Bassham cycle; HM, H_2_ metabolism. (B) Distribution of the carbon and hydrogen metabolic genes in prokaryotic phyla. TPM, transcripts per million. Details of the genes are provided in supplementary data (Tables S9–S11).

**Figure 5:**
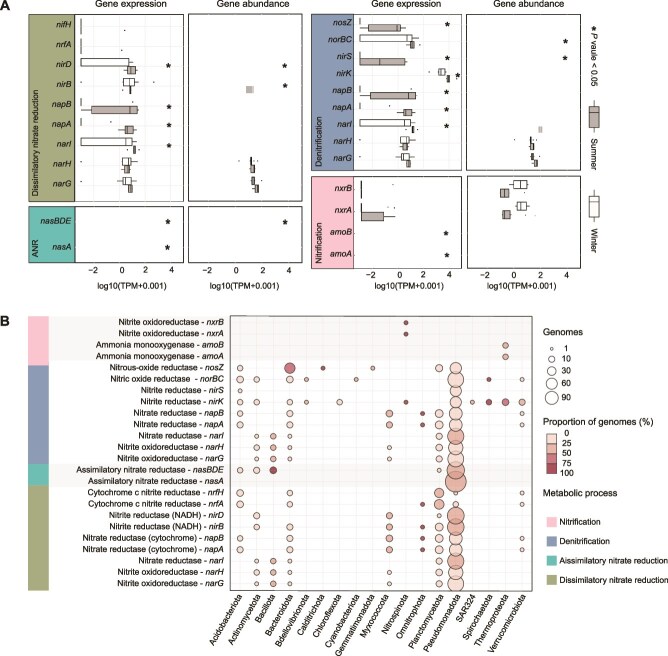
Nitrogen metabolic genes in the metagenomes and metatranscriptomes of the two seasons and their distribution in phyla. (A) TPM of the genes at the genomic and transcriptional levels in the summer and winter samples. All TPM values of the genes mapped by the metagenomic and metatranscriptomic reads have been normalized by mOTUs and transformed using log10(TPM + 0.001). To assess the significance of the differences in TPM values for the same genes between the winter and the summer, the Wilcoxon test for independent samples was performed. The genes showing a *P*-value ≤.05 are marked by a star. ANR, assimilatory nitrate reduction. (B) Distribution of the nitrogen metabolic genes in prokaryotic phyla. TPM, transcripts per million. Comprehensive data for these metabolic genes are available in the supplementary material (Tables S9–S11).

The functional genes identified in the MAGs were utilized to understand the potential lifestyles employed by microorganisms in the sampling environment. The existence and transcription of crucial metabolic genes in unassembled reads were used to identify dominant metabolic pathways. To explore the potential metabolic differences between summer and winter prokaryotic communities, marker genes related to potentially relevant pathways involving carbon metabolism (including methane and CO oxidation, benzoate degradation, carbon fixation, and fatty acid metabolism), nitrogen metabolism, hydrogen oxidation, and environmental information processing were investigated in the deep ocean metagenomic (Figs 4A, 5A, and  S8, Table S8) and metatranscriptomic datasets (Figs 4A and 5A, Tables S10 and S11). To identify the primary disparities in gene expression among distinct phyla, we showed the percentages of various metabolic genes across the MAGs within each respective phylum (Figs 4B and 5B, Table S11). To assess the consistency of replicate samples across seasons, we also performed PCoA based on the TPM values of genes involved in ammonia oxidation, denitrification, carbon monoxide oxidation, and housekeeping genes (40 conserved proteins). The results showed that most of the samples collected in the same season clustered together, indicating high reproducibility in gene expression patterns among replicates (Fig. S9).

### Distinct carbon sources for the deep-sea microorganisms between the seasons

Among the carbon fixation genes associated with the 3-hydroxypropionate/4-hydroxybutyrate cycle (3HP/4HB), the essential gene *4hbd* (K14534) and *hpcs* (K18594) encoded by the phylum *Thermoproteota* (including previous MGI represented here by *Candidatus* Nitrosopelagicus) were significantly enriched in the winter metagenomes than the summer counterparts (Wilcoxon test, *P* ≤ .05; Fig. 4A). In addition to *Thermoproteota*, *4hbd* was also detected in *Proteobacteria*, *Actinobacteria*, *Bacteroidota*, *Acidobacteriota*, and *Myxococcota*, which include noncarbon-fixing organisms. We then calculated the transcripts per million (TPM) of the essential carbon fixation genes of the CBB pathway, *rbcL* (K01601) and *PRK* (K00855), using the metagenomic data. The result showed significantly high relative abundance of the two genes in the summer metagenomic data (Wilcoxon test; *P* ≤ .05; Fig. 4A). The metagenomic and metatranscriptomic reads of *rbcL* and *PRK* genes were affiliated with *Gammaproteobacteria* and SAR324 (Fig. 4B).

Fatty acids can act as a carbon source for some marine microorganisms [51]. The genes *fadA*, *fadB*, *fadJ*, and *fadN* are involved in the β-oxidation pathway of fatty acid catabolism [52]. These enzymes facilitate the degradation of fatty acids into acetyl-CoA, which is subsequently channeled into the TCA cycle or utilized for the biosynthesis of other metabolites. The expression of *fadJ* in the summer was significantly higher than in the winter samples (Wilcoxon test; *P* ≤ .05). TPM values of *fadA* and *fadN* were more than 3-fold higher in the summer than in the winter samples. The genes *fadA*, *fadJ*, and *fadN* were identified in MAGs affiliated with *Acidobacteriota*, *Actinomycetota*, *Bacteroidota*, *Calditrichota*, *Chloroflexota*, *Gemmatimonadota*, *Marinisomatota*, *Myxococcota*, and *Pseudomonadota* (Fig. 4B).

As a complex organic carbon source, benzoate broadens the range of carbon sources available to marine microorganisms [53]. Aromatic compounds consisting of the homocyclic (e.g. benzoate) aromatic nucleus can be metabolized by microorganisms under anaerobic conditions [54]. The genes *dmpL* and *dmpN*, both belonging to the phenol 2-monooxygenase (NADH-dependent) family, facilitate benzoate degradation by converting phenol to catechol. The expression of *dmpL* and *dmpN* associated with Pseudomonadota was significantly higher in the summer (Wilcoxon test; *P ≤ .05*), compared to the winter samples. The genes *mhpA*, *mhpD*, *mhpE*, and *mhpF* are involved in the degradation of aromatic compounds and contribute to the conversion of trans-cinnamate to acetyl-CoA [55]. Our results showed significantly higher expression of *mhpA*, *mhpD*, *mhpE*, and *mhpF* in the summer, compared to the winter (Wilcoxon test; *P* ≤ .05). All the four genes were identified in the MAGs affiliated with *Acidobacteriota*, *Actinomycetota*, *Bacteroidota*, and *Pseudomonadota*. Particulate methane monooxygenase (pMMO) is a membrane-bound enzyme that catalyzes the oxidation of methane to methanol [56]. For the *pmoAB* genes encoding pMMO subunits in our MAGs, the expression of *pmoB* was higher in the summer than in the winter with a > 30-fold increase (Fig. 4A). The marker genes associated with methane oxidation and subsequent assimilation, including *pmoA*, *pmoB*, *xoxF*, and *mdh1*, were detected exclusively in *Pseudomonadota* (Fig. 4B).

### Prevalence of autotrophic deep-sea ammonia-oxidizing archaea in winter

Autotrophic microorganisms involved in processes such as ammonia oxidation have been regarded as the main energy providers in the deep ocean [57–59]. We investigated the expression level of the ammonia oxidation marker genes coding for ammonia monooxygenase, which showed significantly higher transcriptional level of *amoA* and *amoB* genes in the winter, compared to the summer (Wilcoxon test; *P* ≤ .05; Fig. 5A). Almost all of the *amoA* and *amoB* genes were affiliated with known ammonia-oxidizing archaea (AOA), *Nitrosopumilaceae* (*Thermoproteota*) (Fig. 5B). AOA rely on the 3HP/4HB cycle for carbon fixation. Therefore, we compared the TPM values of the marker genes *4hbd* and *hpcs* of the 3HP/4HB pathway in the MAGs classified under the order *Nitrososphaerales*. The results showed significantly higher expression of both genes in the winter, compared with the summer samples (Wilcoxon test, *P* ≤ .05; Fig. S10).

### Denitrification genes facilitate higher energy acquisition in summer

Denitrification is an important metabolic pathway for microorganisms to obtain energy through nitrate respiration in anoxic environment [60]. To investigate the seasonal dynamics of denitrification, we compared the expression levels of all the denitrification associated genes between the summer and winter samples. Transcription of all the genes was elevated in the summer, with *nosZ*, *nirS*, *nirK*, *narI*, *napA*, and *napB* showing significantly higher expression, compared to the winter (Wilcoxon test, *P* ≤ .05). The genes *nosZ*, *norBC*, *nirK*, *narI*, *napA, napB, narH*, and *narG* were all possessed by the phyla *Bacteroidota* and *Pseudomonadota*. The *nosZ*, *norBC*, *nirK*, *narI*, *napA*, and *napB* genes were associated with *Acidobacteriota* (Fig. 5B).

### Differential expression of hydrogen and carbon monoxide oxidation genes between summer and winter

H_2_ oxidation is an important energy source for marine microorganisms [43]. Using curated hydrogenase sequences from a published dataset [43], we constructed a reference database and performed sequence alignment. Our analysis revealed that NiFe hydrogenases exhibited significantly higher transcriptional activity in the summer, compared to the winter (Wilcoxon test, *P* ≤ .05), whereas FeFe hydrogenase genes showed no detectable transcriptional activity in either season (Fig. 4A). The NiFe hydrogenases were primarily affiliated with the phyla *Bacillota*, *Cyanobacteriota*, *Gemmatimonadota*, and *Pseudomonadota* (Fig. 4B). We further analyzed the seasonal expression patterns of NiFe hydrogenases in *Burkholderiales*, as this order exhibited the highest relative abundance of these genes. Results showed that NiFe hydrogenase expression in the summer was significantly higher than that in the winter (Wilcoxon test, *P* ≤ .05; Fig. S10). In addition to hydrogen, some archaea and bacteria can utilize carbon monoxide (CO) as an alternative energy source [61]. We analyzed the seasonal expression patterns of CO dehydrogenase genes (*coxSML*) and found that *coxS* was expressed at a significantly higher level in the winter than in the summer (Wilcoxon test, *P* ≤ 0.05). The MAGs from multiple phyla, including *Acidobacteriota*, *Actinomycetota*, *Bacteroidota*, *Chloroflexota*, *Gemmatimonadota*, *Marinisomatota*, *Myxococcota*, and *Pseudomonadota*, contained *coxSML* genes. We further analyzed the seasonal expression patterns of *coxSML* in *Marinisomatales*, as this order contained the majority of CO dehydrogenase genes. As expected, the expression of *coxM* and *coxL* genes in the winter was significantly higher than in summer (Wilcoxon test, *P* ≤ .05; Fig. S10).

### Higher gene expression of environmental information processing in summer

In addition to the carbon, nitrogen, and hydrogen metabolism genes, seasonal variants in transcriptional level between the seasons were displayed in the two-component genes *pilU*, *pilR*, *pilQ*, *pilP*, *dctD*, and *dctB* (Fig. S11 and Tables S9–S11). All these genes were significantly higher expressed in the summer than the winter (Wilcoxon test, *P* ≤ .05). Among them, *dctD* and *dctB* are components of the dicarboxylate transport (Dct) system, which facilitates the uptake, exchange, and efflux of C4-dicarboxylates [62]. The genes *pilU*, *pilR*, *pilO*, and *pilP* are associated with type IV pilus assembly and motility [63].

### Co-occurrence pattern varies in the prokaryotic communities between two seasons

**Figure 6:**
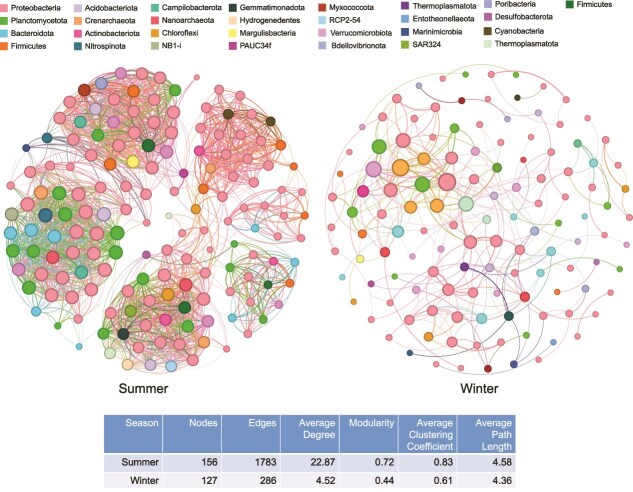
Co-occurrence network of prokaryotic communities based on 16S rRNA gene sequences in the metatranscriptomes for the summer and winter samples. Only nodes (OTUs) that were significantly correlated with each other (Spearman’s correlation > 0.8; after Benjamini and Hochberg FDR adjustment, *P* < .05) were connected (edges). The nodes representing the taxa from the same phyla in the network are shown with the same color. The size of the nodes is proportional to the number of connections. The edge thickness indicates the correlation values. For specific parameters of the constructed network, refer to Tables S12 and S13.

The prokaryotic network of the communities based on the 16S rRNA gene sequences of metatranscriptomes demonstrated distinct co-occurrence patterns between the summer and winter. The microbial network complexity was assessed by using the network topological parameters composed of node and edge numbers, average degree, modularity, average clustering coefficient, and average path length (Figs 6, S12, and  S13). The prokaryotic network in the summer was more complex, as indicated by higher node and edge numbers in the summer than in winter. According to the ranking of the top 11 prokaryotes in terms of the average degree in the winter, 3 of them were AOA (*Candidatus* Nitrosopumilus, *Nitrosopumilaceae*, and *Candidatus* Nitrosotenuis) (Tables S12 and S13). High mean degree, high closeness centrality, and low betweenness centrality can be used collectively to identify keystone taxa [64, 65]. OTUs with a degree of >6 and > 20 for the winter and summer data, respectively, betweenness centrality <100, and clustering coefficient > 0.4 were selected as potential keystone taxa. In summer, these OTUs were mainly affiliated to *Proteobacteria*, *Crenarchaeota*, and *Actinobacteriota*, whereas in winter, they were affiliated with *Proteobacteria*, *Actinobacteriota*, *Bacteroidota*, and *Planctomycetota*.

## Discussion

### Seasonal genomic shifts with stable transcriptional profiles in deep-sea microbial communities

With the MISNAC sampling method [17], we report the *in situ* community structures and temporal transcriptomes of active microorganisms in the deep-sea sites of SCS. Our PCoA results of prokaryotic microbial communities in the metagenomes revealed a substantial separation and a high dissimilarity between the communities of prokaryotic microbiomes in the summer and winter stations. In contrast, the 16S rRNA gene reads extracted from metatranscriptomic data between the winter and summer exhibited a high degree of similarity, indicating that at the transcriptional level, prokaryotic activity between winter and summer exhibited low variability. This suggests limited variation of active deep-sea inhabitants. The community structural differences between the seasons based on metagenomic survey are probably attributed to the distinct microbial taxa derived from the ocean surface, especially during summer with high primary production as indicated elsewhere [4, 66]. These microorganisms likely attach to “marine snow” and subsequently descend to deeper layers. In our study, the top 10 MAGs with the highest relative coverage in metagenomes included particle-attached microorganisms commonly found in marine environments, such as *Sphingomonadaceae* [67] (*Novosphingobium meiothermophilum*) and *Burkholderiaceae* [68] (*Aquabacterium, Methyloversatilis*, *Azospira oryzae*) in summer, along with *Sphingomonadaceae* [67] (*Sphingobium yanoikuyae*), *Moraxellaceae* [69] (*Acinetobacter junii*), *Parvularculaceae* [69] (*Marinicaulis*), and *Rhodobacteraceae* [70] (*Pseudooceanicola*) in winter. The seasonal variations in communities are likely ascribed to vertical convection during winter overturning [11]. These organisms were dominant in the 16S rRNA gene-based communities but showed a low transcriptional level.

Deep-sea microbial transcriptomes are rarely reported, particularly with respect to identifying active microorganisms through metatranscriptomic read coverage of MAGs. Metatranscriptomes were dominated by *Gammaproteobacteria* (*Methylomonadaceae* and *Burkholderiales*), *Alphaproteobacteria* (AEGEAN-169 marine group and SAR11 Clade II), and *Marinimicrobia* as determined from 16S rRNA gene sequences. *Gammaproteobacteria* and *Alphaproteobacteria* have also been reported in other studies of deep-sea waters in SCS [71]. In the North Pacific Subtropical Gyre of 4000 m depth, *Arcobacter* (*Campylobacterota*) and *Colwellia* (*Gammaproteobacteria*) were the most actively transcribed prokaryotic genera in the marine snow particles [72]; however, they were not among the most active microorganisms in this study. Based on the relative abundance of MAGs in the metatranscriptomes, the transcriptionally active prokaryotic groups in summer included *Nitrososphaeria*, *Poseidoniia*, SAR324, *Gammaproteobacteria*, and *Marinisomatia*. These MAGs were also highly active in winter, suggesting that the dominant active prokaryotic taxa in the deep SCS are consistent across seasons.

### Ciliophora and *Retaria* can act as key energy transmitters in the deep-sea food chain

In our dataset, *Ciliophora* and *Cercozoa* displayed higher activity than *Retaria*, *Euglenozoa*, and *Protalveolata*, which were the most abundant lineages in the 18S rRNA gene sequences of the metagenomes. A similar phenomenon has been demonstrated in the marine snow particles [72]. The abundance and activity of eukaryotic organisms varied greatly in the time-course metatranscriptomes, with clear transcriptomic differences between time intervals and seasons. This indicates high functional diversity among deep-sea eukaryotic microorganisms [73, 74]. *Ciliophora* have rarely been reported as important members of the microbial community at 1000 m depth and have more rarely been shown to exhibit seasonal patterns [72]. However, *Ciliophora* revealed the highest transcriptional activity among eukaryotes in our samples, likely playing a major role as a primary consumer between 1022 and 1084 m in the summer sampling period, contributing to the transfer of energy and nutrients within the aquatic ecosystem. The high transcriptional activity of *Ciliophora* in the summer indicates that they likely act as an important food and energy source for higher trophic levels such as planktonic copepods [75, 76].

*Retaria* was the most abundant eukaryotic phylum in our metagenomic datasets. In the California Current Ecosystem, *Retaria* was also identified as the most abundant group in particulate organic matter (POM) sinking out of the euphotic zone. Phytoplankton and metazoan zooplankton are usually considered to play a major role in vertical export [77]. Although Radiolarians are closely involved in the biogeochemical cycles of carbon and transport large amounts of organic matter to the deep ocean [78, 79], our metatranscriptomics result suggests weak activity of the Radiolarians. Nevertheless, the higher abundance of Radiolarians in the summer samples suggests more descending radiolarian necromass, which may serve as a potential energy source and habitat for heterotrophic microorganisms in the deep ocean.

### Seasonal shifts in energy utilization by deep-sea microorganisms

Key biogeochemical functions of marine surface microorganisms revealed seasonal variation. Analysis of metagenomic data in Blanes Bay surface seawater revealed that genes related to organic matter synthesis and energy production, such as biosynthesis of the photosynthetic-related gene *pufML*, carbon monoxide oxidizer *coxL*, and taurine transporter *tauA*, as well as the phosphorous cycling genes were enriched during spring and summer, while *amoA* was most abundant in autumn and winter [66]. Primary production in the summer ocean surface is therefore much stronger than in winter, which can export marine POM input into the deep sea. POM in the marine snow is comprised of decaying phytoplankton and faecal matter generated by zooplankton, as well as aggregates of high-molecular-weight dissolved organic matter [77, 80], which provides abundant food for its associated microorganisms [80, 81]. In a previous expedition, a Lagrangian Particle Tracking Model (LPTM) was employed to trace the sources and transport pathways of sinking particles at a depth of 1000 m in the northwestern SCS near our sampling location [82]. Time-series analyses of diatom, biogenic opal, and organic matter fluxes showed higher concentrations in July, compared to November [82]. Sediment trap observations and LPTM results indicated that the seasonal variation may be affected by resuspended sediment associated with coastal upwelling driven by the summer monsoon [82]. In addition to laterally transported resuspended sediments, organic matter originating from the ocean surface may also represent a substantial contributor to POM. Previous studies have investigated seasonal patterns of marine net primary production (NPP) and reported that NPP was higher in summer than in winter during the period of 2002–20 at the location of our sampling sites [83]. Similarly, dissolved organic carbon (DOC) and particulate organic carbon (POC) concentrations in surface waters near our study sites were higher in summer than in other seasons [84]. Thus, a higher proportion of organic debris is expected at deep-sea sites during summer, which is consistent with our transcriptomics results that showed elevated expression levels of genes involved in fatty acid metabolism and benzoate degradation in summer.

Surface ocean microorganisms can be transported to the deep ocean via vertical water exchange and sinking particles. To assess this potential connection, we also analyzed surface water prokaryotic communities using publicly available data [28]. Several taxa inhabiting the surface water adjacent to our sampling stations also exhibit high abundance in our deep-sea samples, further supporting the potential link between surface and deep-sea microbial communities. However, most of the dominating prokaryotic species on the surface exhibited no detectable activity in the deep-sea environment, suggesting their surface origin and inactivity. In winter, organic matter biosynthesis in the marine surface decreases, followed by a lower concentration of POM flux to the deep sea. Additionally, the contribution of resuspended sediments is minimal in winter, further reducing the POM flux. As a result, deep-sea autotrophic microorganisms may dominate and contribute organic carbon to sustain this ecosystem. This interpretation is supported by the observed high expression of ammonia oxidation and 3HP/4HB pathways, as well as by the winter microbial network being centered on AOA. An alternative explanation is that ammonia-based new production is higher in winter than in summer in the SCS [85]. During winter, the northeast monsoon may transport nitrate nitrogen from the East China Sea into the SCS [86], which might provide an additional amount of nitrate as a nitrogen source for the deep-sea microbiota.

Plentiful marine POM sinking to ~1000 m during summer provides an ample supply of organic material, making microorganisms less reliant on energy-intensive organic synthesis. In our dataset, energy in summer primarily appeared to originate from heterotrophic microorganisms capable of fatty acid degradation, methane oxidation, benzoate degradation, denitrification, and H_2_ oxidation to sustain their growth in the summer. In addition, several genes associated with two-component systems showed significantly higher expression levels in summer than in winter. For instance, *dctD* and *dctB*, which are components of the dicarboxylate transport (Dct) system responsible for the uptake, exchange, and efflux of C4-dicarboxylates, displayed seasonal upregulation, likely linked to the increased deposition of organic matter in the deep sea during summer [62]. Similarly, several genes involved in pilus assembly and motility, including *pilU*, *pilR*, *pilO*, and *pilP*, also showed elevated expression in the summer. The enhanced expression of these genes might promote the synthesis and twitching motility of type IV pili, enabling bacteria to actively search for and access nutrient sources in response to the seasonal increase in organic matter [63]. Denitrification is a respiratory process that typically occurs under anaerobic conditions in facultative bacteria, whereby nitrate is sequentially reduced to nitrite, NO, N_2_O, and finally to N_2_ [87]. Denitrification marker genes such as *napA*, *norB*, and *nosZ* have been reported as enriched in mesopelagic metatranscriptomes from the Tara Ocean Project [50]. In our research, the high expression of denitrification genes in summer transcriptomes suggests possible suboxic or anoxic microenvironments within organic particles [4], even though the surrounding seawater at the sampling depth was oxygenated [88]. This interpretation is consistent with reports that CO and ammonia oxidation were prevalent in free-living microbial communities, whereas H_2_ oxidation and dissimilatory nitrate reduction to ammonium (DNRA) were more abundant in particle-attached microbial communities [4]. In line with this, we observed that particle-associated processes, such as H_2_ oxidation and DNRA, exhibited higher activity in summer, likely driven by the increased availability of organic particles. In contrast, CO and ammonia oxidation, more typical of free-living microorganisms, were more prominent in winter when particle flux was lower [4, 82].

### Nutrient source variations might influence the microbial network structure

Our results showed that microbiome complexity and keystone taxa differed between summer and winter samples. Network analysis revealed higher complexity in summer as indicated by a larger number of nodes and edges compared with winter. This may be due to the more diverse nutritional sources available to summer deep-sea microbiomes, which promote more active metabolism and thus greater network complexity, as reflected by the higher average coverage of MAGs in the summer metatranscriptomic data. POM is heterogeneous, with different particles containing varying compositions [89], and microorganisms show preferences for different particle types [90]. These preferences may explain the modular structure of the summer network, which consisted of six distinct modules. In winter, the likely reduced flux of POM to the seafloor had relatively little effect on microbial network organization. AOA emerged as core taxa because their activity supplies labile organic carbon through ammonia oxidation. Thus, although AOA (*Nitrososphaeria*) were relatively low in abundance during summer, they were still identified as keystone taxa, a category defined as species that exert a disproportionately large effect on the ecosystem relative to their abundance [91]. In contrast, in winter, their increased abundance and high transcriptional activity further underscored their essential role as core members of the network. Collectively, the seasonal shift in nutrient regimes likely drove the observed differences in microbial network structure.

## Conclusion

Overall, our new sampling strategy provides improved capacity to characterize community structures and potential functions of active deep-sea microorganisms, by enabling time-series collections with minimal recovery bias. We revealed pronounced seasonal changes in the metabolic functions and ecological strategies of deep-sea microorganisms, providing an approach to better understanding deep-sea biogeochemical processes mediated by resident microbial communities. Such insights are critical for quantifying and modeling the carbon cycle in the deep ocean. Although the MISNAC method improves *in situ* fidelity over traditional approaches, prolonged filtration may alter gene expression profiles. In addition, enrichment of microbial predators such as “ciliates” during filtration may have altered the community structure. To address these limitations, future studies should compare different *in situ* sampling methods to evaluate their effects on microbial gene expression and community composition. With additional time-course MISNAC collections from different depths and sites globally, it will be possible to gain more mechanistic insights into carbon cycling across scales in the deep ocean.

## Supplementary Material

20250913_Supplementary_figures_wraf214

20250913_Supplementary_Tables_wraf214

## Data Availability

All data are available in the main text or the supplementary materials. The raw metatranscriptome sequencing data, assembled contigs, and MAG sequences generated in this study have been deposited in the NCBI database under the accession code PRJNA1067282. We have archived the datasets for amino acid sequences and annotation of MAGs in the Zenodo repository. The datasets are accessible through the following Digital Object Identifier (DOI): https://zenodo.org/records/15255130. We also uploaded the modified version of the Green Lab metabolic marker protein database and its corresponding annotation file to Zenodo (https://zenodo.org/records/15919177). Furthermore, the analysis codes are publicly available at https://github.com/heyinghui22/Codes-for-Transcriptional-Difference-of-Deep-Sea-Microorganisms-Under-Different-Sampling-Methods.
