# Supplementary material for: Seasonal variability in community structure and metabolism of active deep-sea microorganisms
Source: ISME J. 2025 Sep 23;19(1):wraf214. doi: 10.1093/ismejo/wraf214 (PMC12507015; doi:10.1093/ismejo/wraf214)
Supplement: 20250913_Supplementary_figures_wraf214 [file 20250913_supplementary_figures_wraf214.docx]

Supplementary Figures for

**Seasonal variability in community structure and metabolism of active deep-sea microorganisms**

Yinghui He^1^, Federico Baltar^2^, Yong Wang^1, 3,*^

^1^Institute for Ocean Engineering, Shenzhen International Graduate School, Tsinghua University, Shenzhen, P.R. of China

^2^College of Oceanography and ecological science, Shanghai Ocean University, Shanghai, P.R. of China

^3^Shenzhen Key Laboratory of Advanced Technology for Marine Ecology, Shenzhen International Graduate School, Tsinghua University, Shenzhen, P.R. of China

^*^Corresponding author:

Yong Wang, PhD

**Email:**  wangyong@sz.tsinghua.edu.cn

# Figure S1.


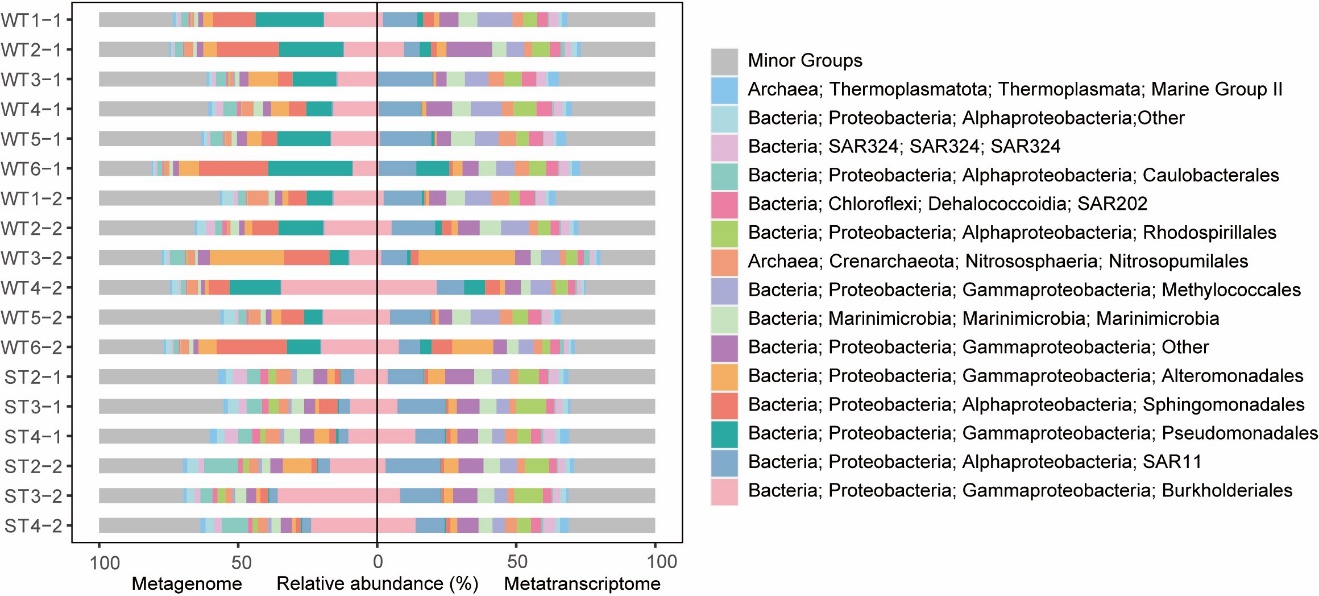


Figure S1. Community structures of prokaryotes at order level.

The relative abundance of prokaryotes at order level was estimated based on analysis of 16S rRNA gene sequences extracted from metagenomes and metatranscriptomes.

# Figure S2.


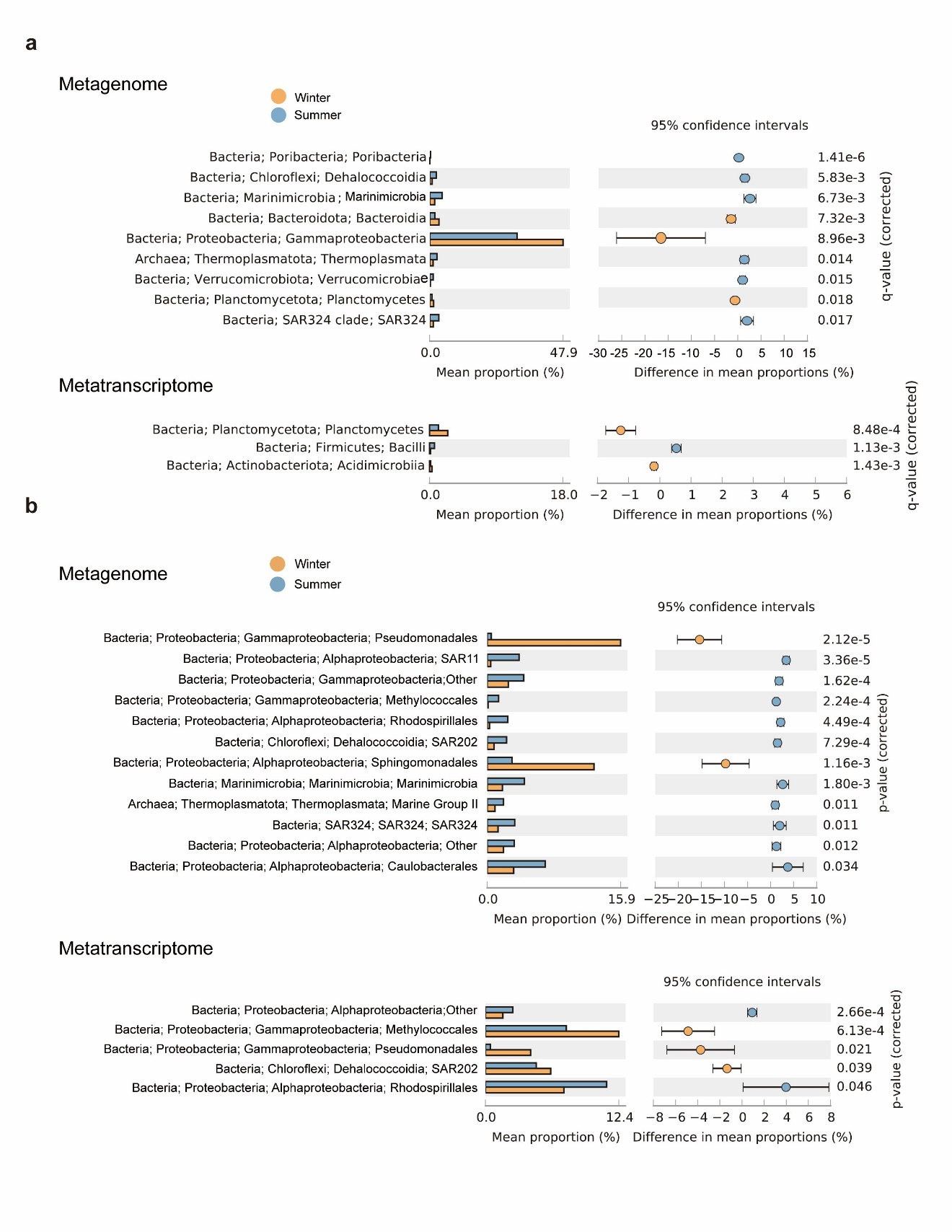


Figure S2. STAMP analysis of prokaryotic community structures at class (a) and order (b) levels.

The classes and orders that exhibit significant differences between the winter and summer were revealed using STAMP analysis. The statistical analysis was performed using a Welch's t-test with Benjamini FDR correction to show the taxa with a significant difference (*P* < 0.05).

# Figure S3.


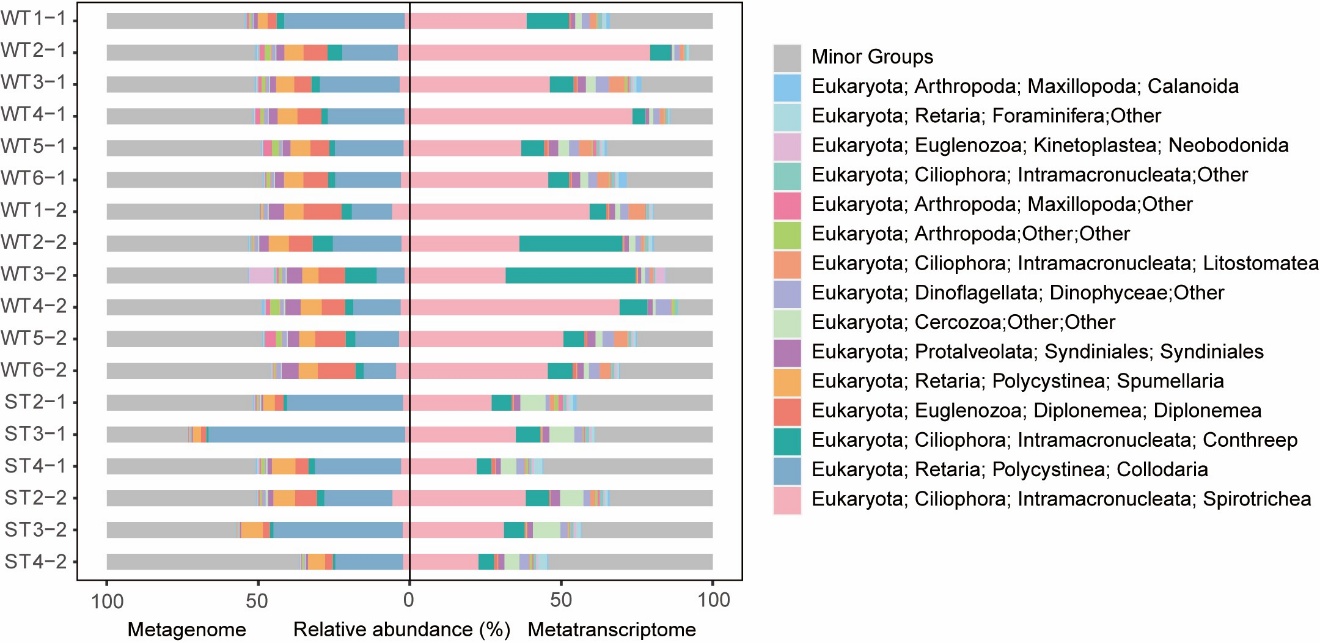


Figure S3. Community structures of eukaryotic microorganisms at order level.

The relative abundance of eukaryotes was based on 18S rRNA gene sequences extracted from metagenomes and metatranscriptomes.

# Figure S4.


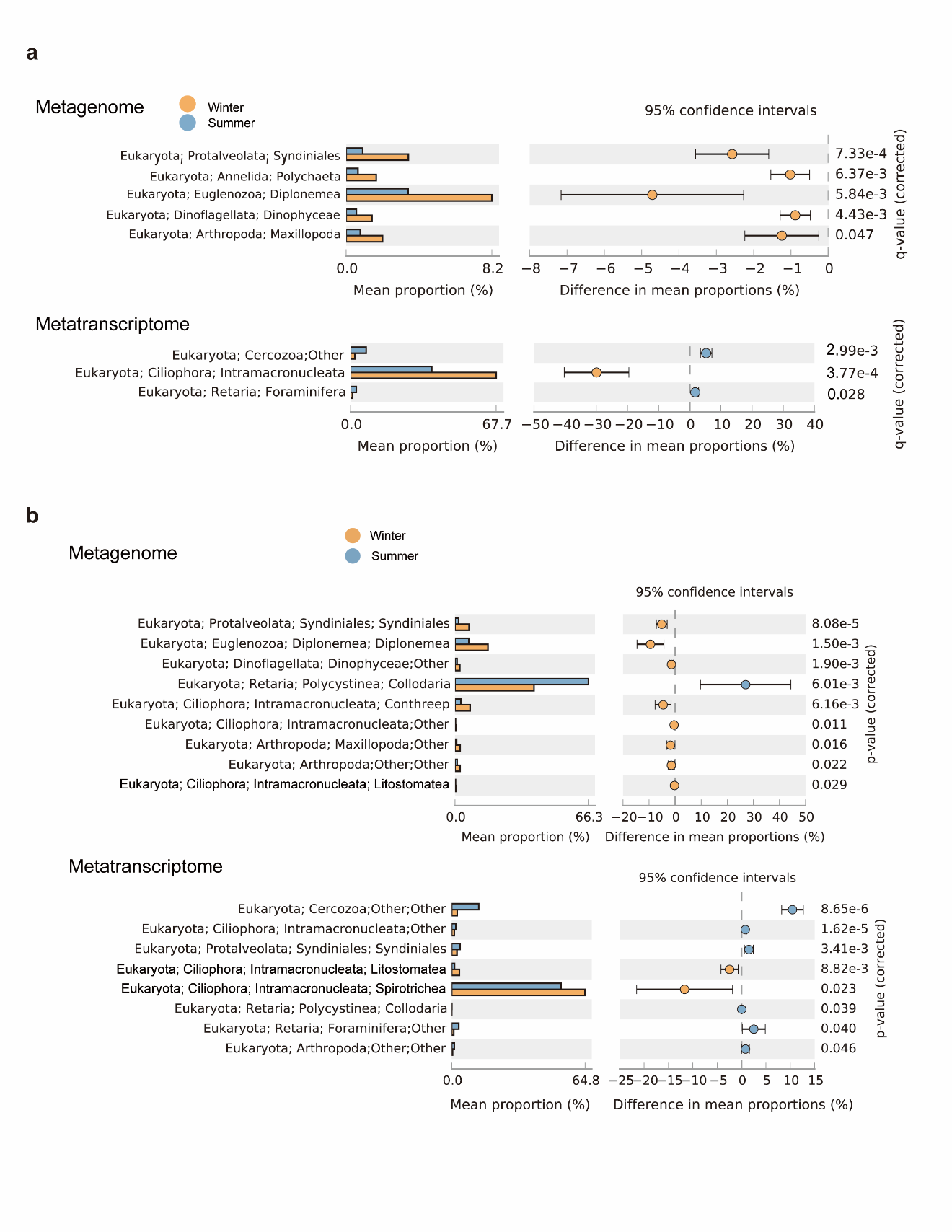


Figure S4. STAMP analysis of eukaryotic microbial community structures at class (a) and order (b) levels.

The classes and orders that exhibit a significant difference between the winter and summer datasets were revealed using STAMP analysis. The statistical analysis was performed using a Welch's t-test with Benjamini FDR correction to show the taxa with a significant difference (*P* < 0.05).

# Figure S5.


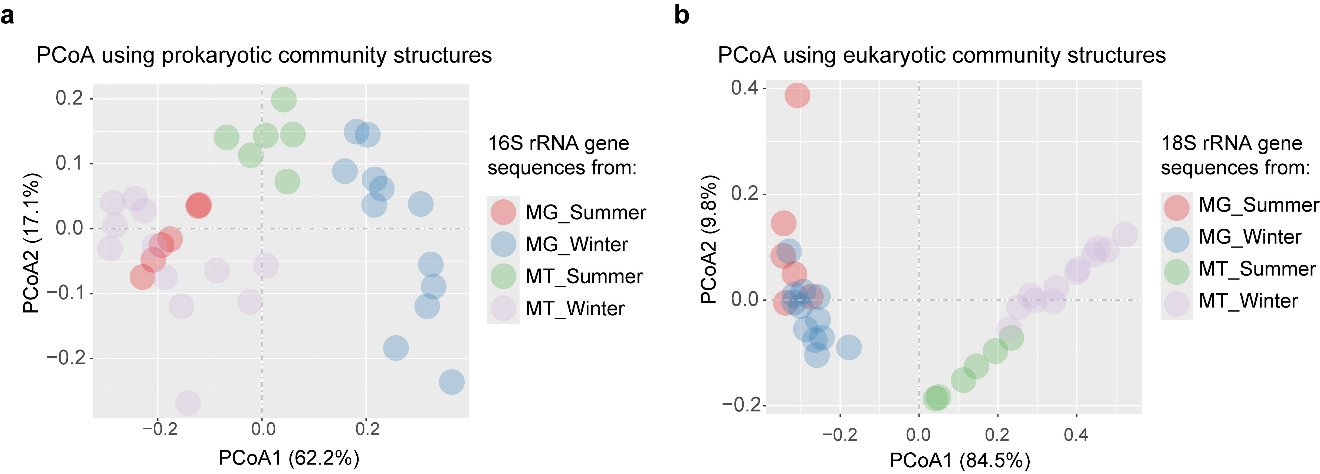


Figure S5. The principal coordinate analysis (PCoA) plots for prokaryotic (a) and eukaryotic (b) community structures.

The plots were based on Bray-Curtis dissimilarity matrices calculated using taxonomic classification results (class level) of 16S and 18S rRNA gene sequences extracted from the metagenomic (MG) and metatranscriptomic (MT) reads.

# Figure S6.


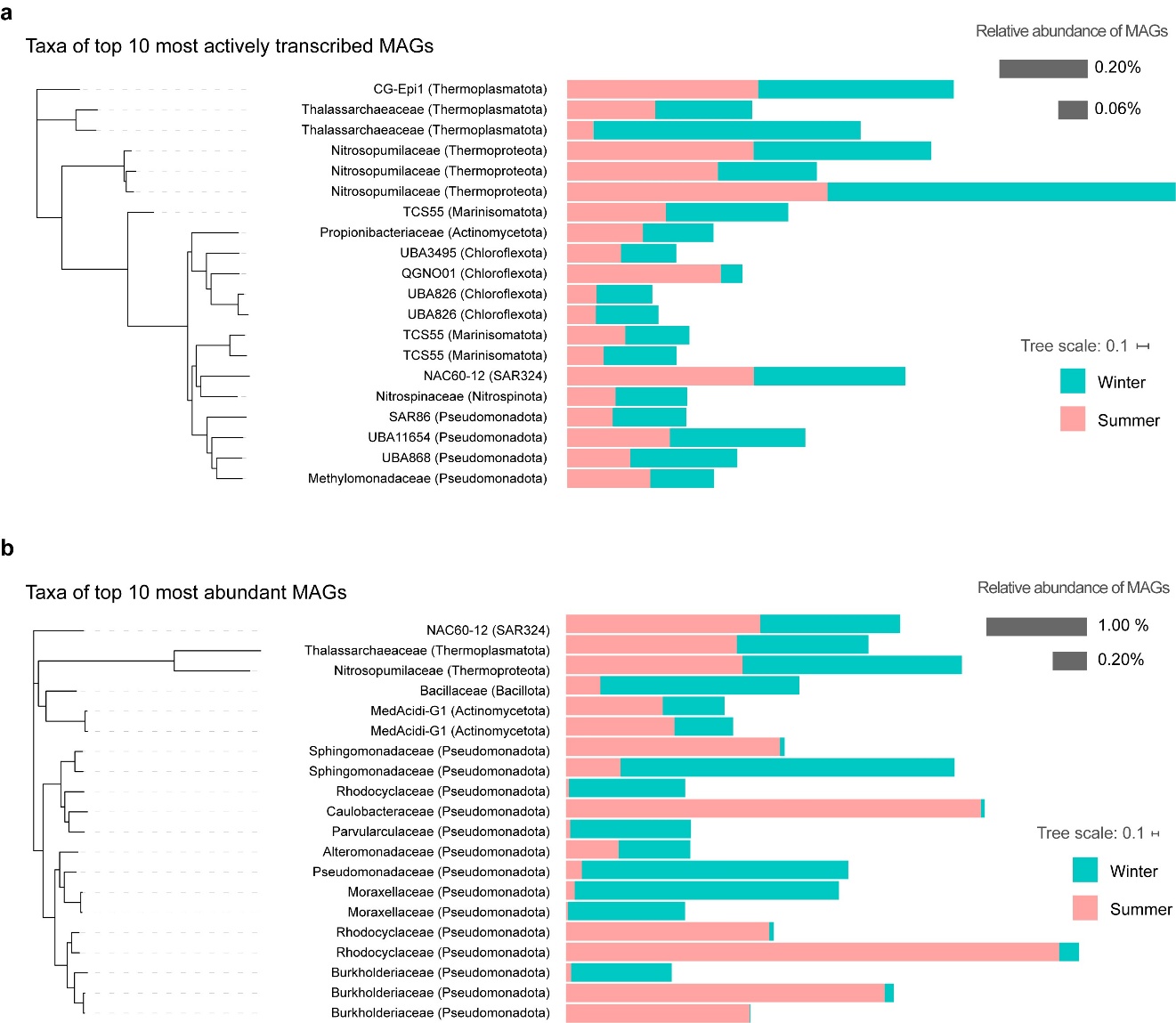


**Figure S6. Seasonal variation in relative abundance of the most transcriptionally active and most abundant species represented by MAGs.**

(a) The relative abundance of the top 10 most active species represented by MAGs. The transcriptional level of the species was estimated by the percentage of the reads recruited by the MAGs from the metatranscriptomes, respectively. The pink bars indicate the read recruitment of each MAG mapped by the summer metatranscriptomes, and the green bars represent that of the MAGs in the winter.

(b) The relative abundance of the top 10 most abundant MAGs based on metagenomic data. The red bars indicate the percentage of the reads recruited by each MAG from the summer metagenomes, while the green bars represent the percentage calculated using the winter metagenomes.

# Figure S7.


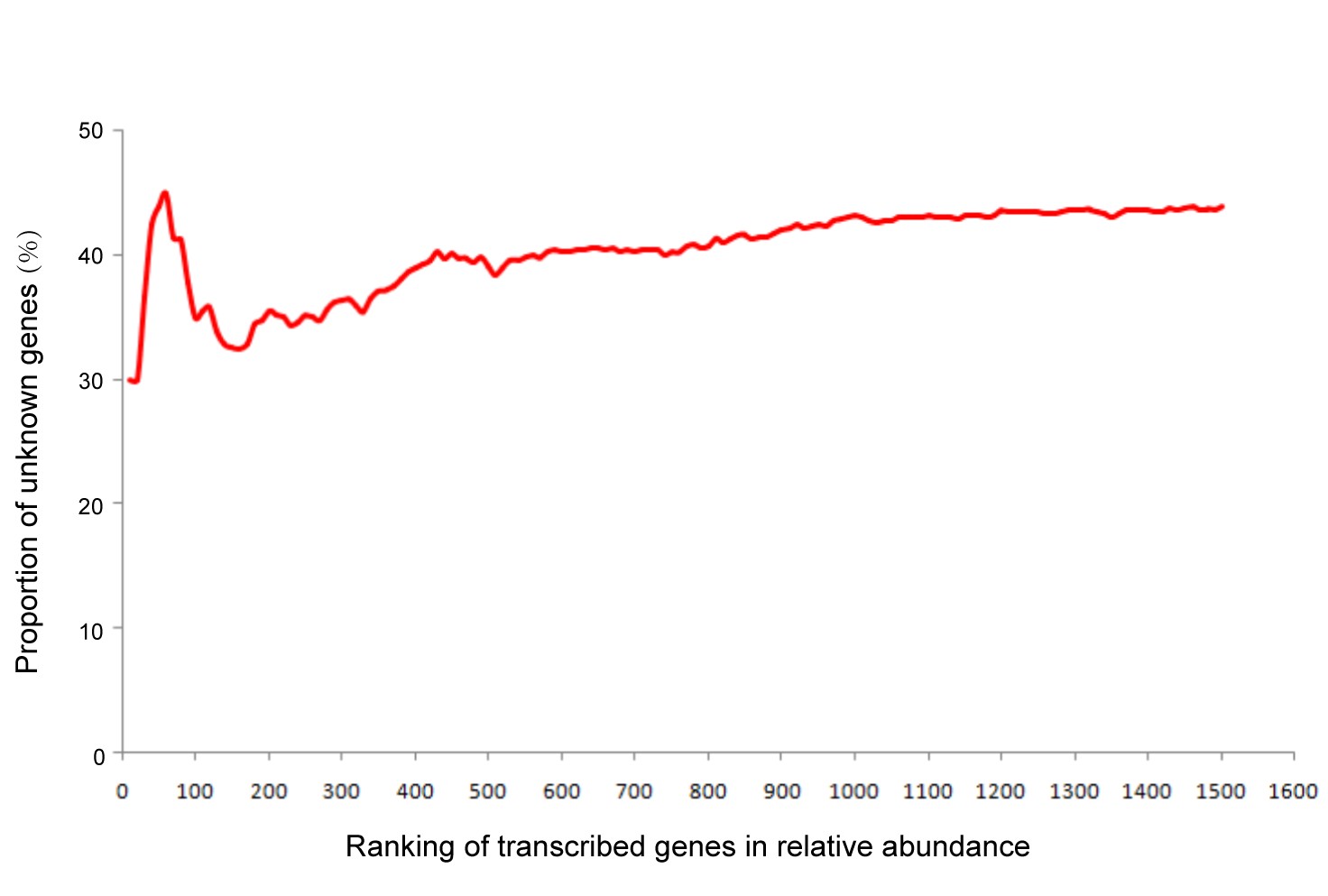


Figure S7. Proportion of unknown genes in highly transcribed genes.

The number of unknown genes in a range of the top transcribed genes ranked in x axis was used to calculate the percentage in y axis.

# Figure S8.


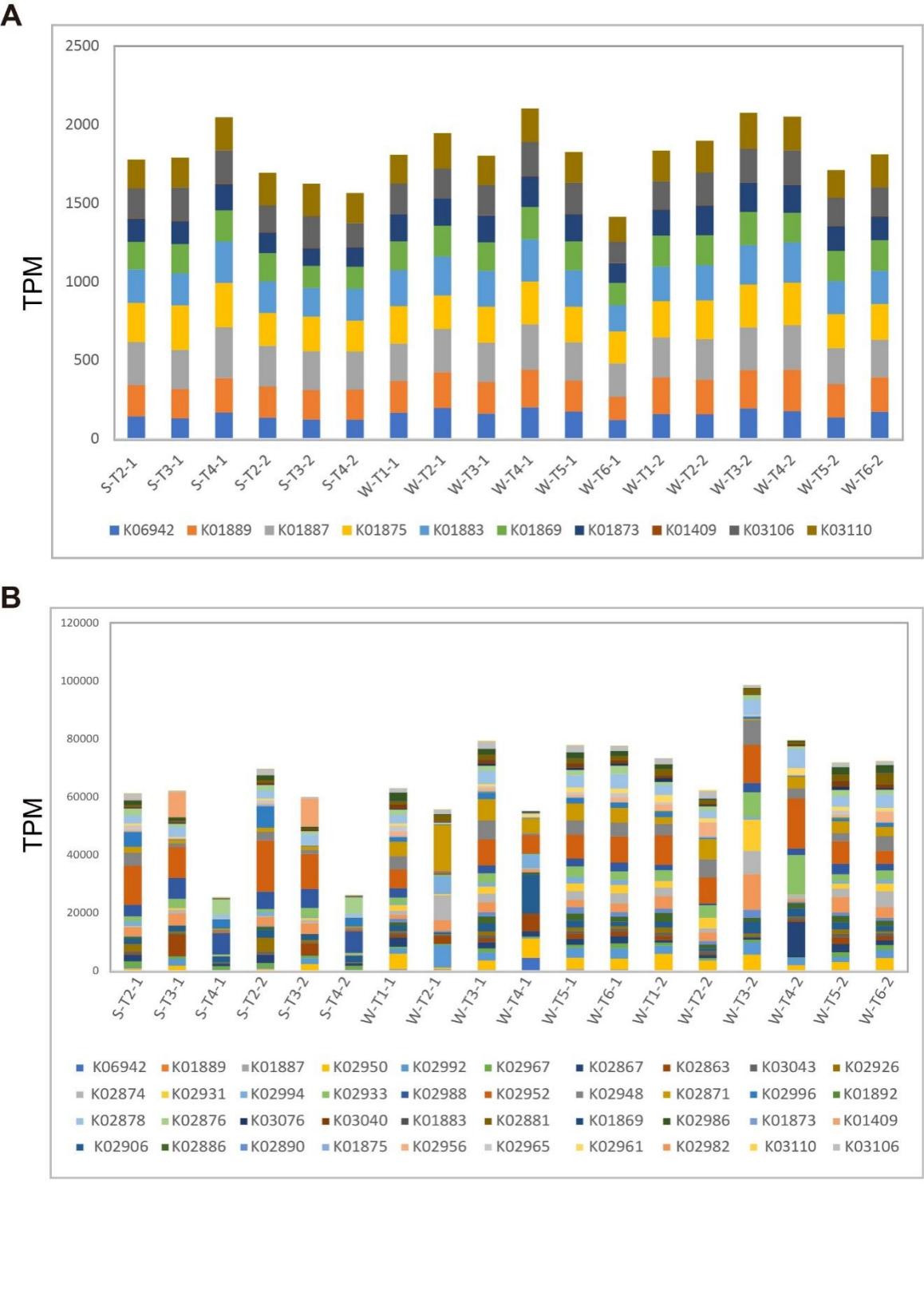


Figure S8. Total coverage of conserved single-copy genes in metagenomes and metatranscriptomes across samples.

The total coverage of 10 conserved single-copy genes in the metagenomes (A) and 40 conserved single-copy genes in the metatranscriptomes (B) were displayed by stacked bar chart. The data were used for normalization of prokaryotic metagenomes and metatranscriptomes between different time-course samples. TPM, total of transcripts per million reads.

# Figure S9.


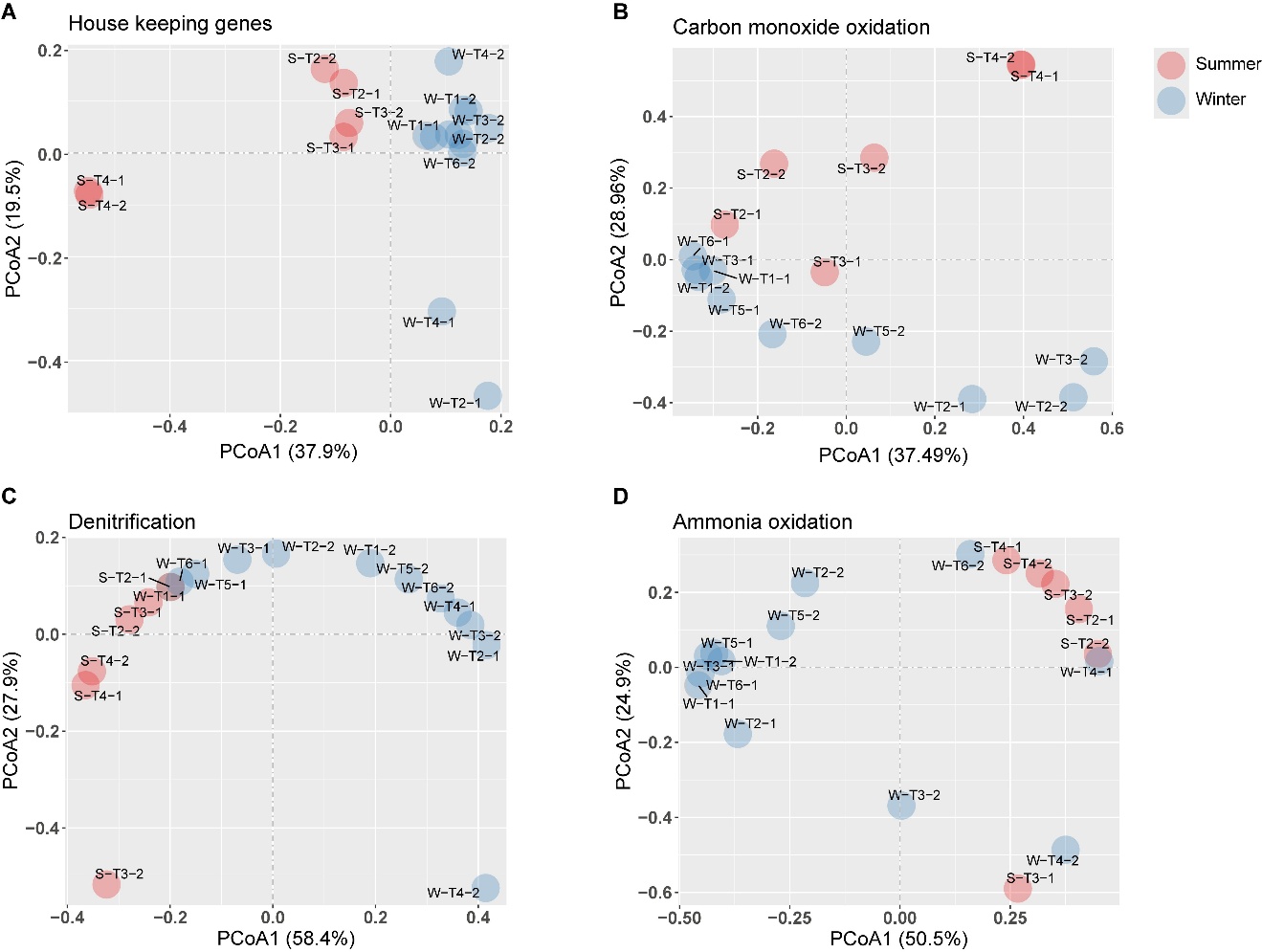


Figure S9. The principal coordinate analysis (PCoA) plots for prokaryotic transcriptional expression pattern across functional genes and housekeeping genes

The plots were based on Bray-Curtis dissimilarity matrices calculated using TPM values of genes involved in housekeeping gene related pathways(A) and functional pathway including carbon monoxide oxidation (B), denitrification(C), and ammonia oxidation pathways. These pathways were chosen for analysis because other functional pathways of interest contained zero values in some samples, preventing their analysis using PCoA.

# Figure S10.


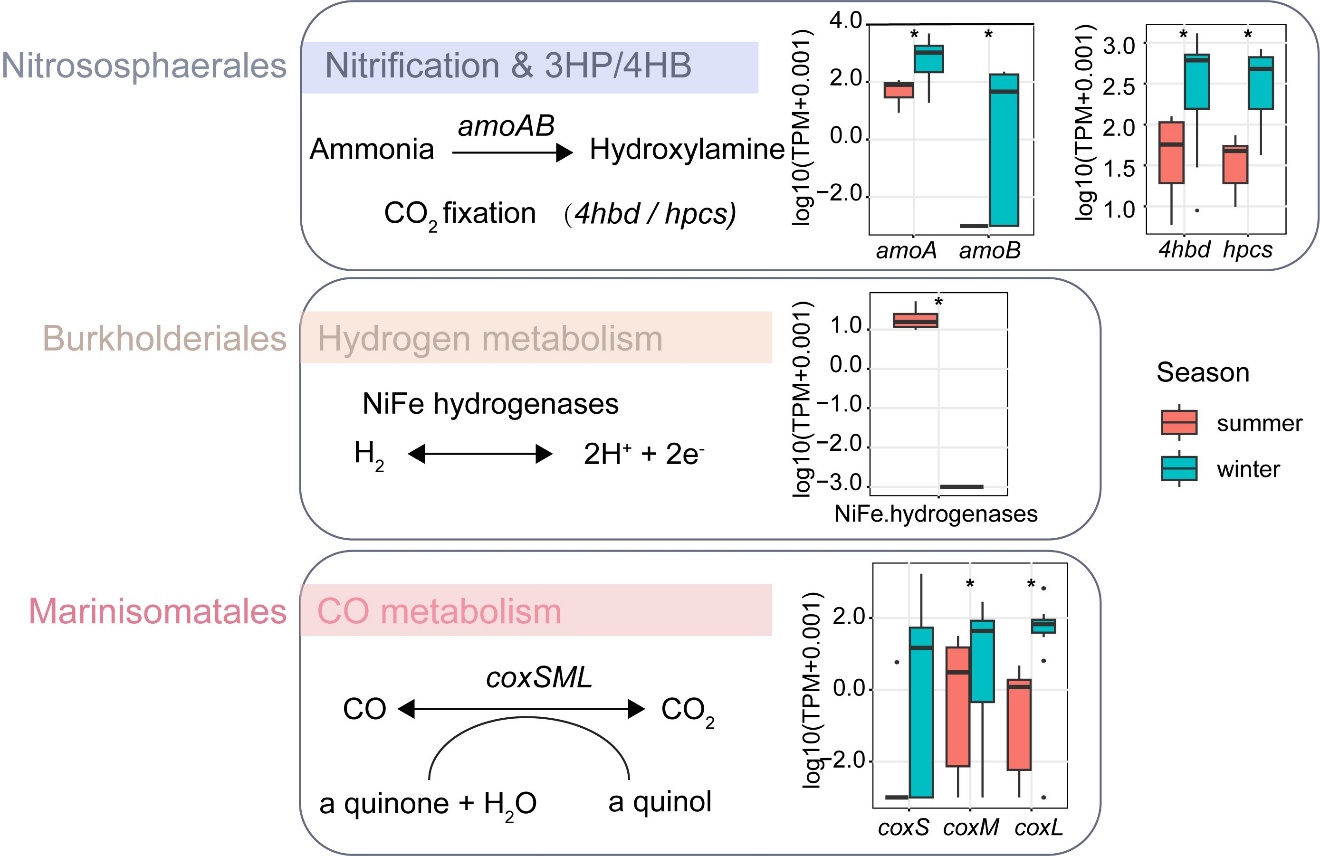


Figure S10. Transcriptional activities of ammonia oxidizing, NiFe hydrogenase and CO oxidizing genes among specific prokaryotic orders between summer and winter.

All values have been transformed using log10 (TPM+0.001). To assess the significance of the differences in TPM values for the same genes between the winter and summer datasets, the Wilcoxon test for independent samples was performed. The genes showing a *P* value less than or equal to 0.05 are marked by a star.

# Figure S11.


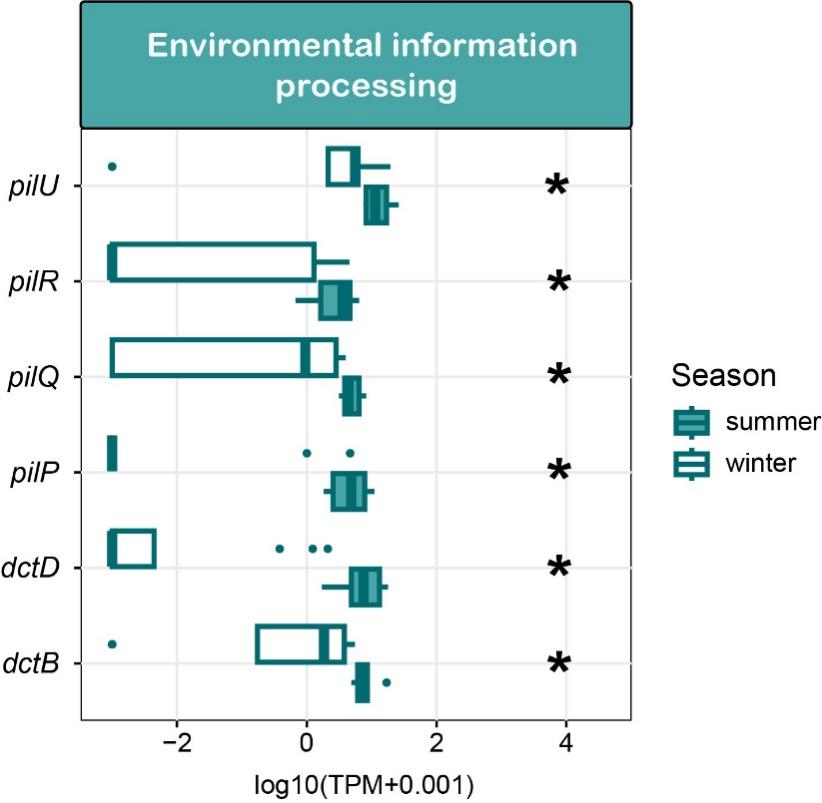


Figure S11. Relative abundance of environmental information processing genes in metatranscriptomic data of the two seasons.

All TPM values of the genes mapped by the metagenomic reads have been normalized and transformed using log10(TPM + 0.001). To assess the significance of the differences in TPM values for the same genes between the winter and the summer, the Wilcoxon test for independent samples was performed. *P* values that are less than or equal to 0.05 were marked by a star in the figure.

# Figure S12.


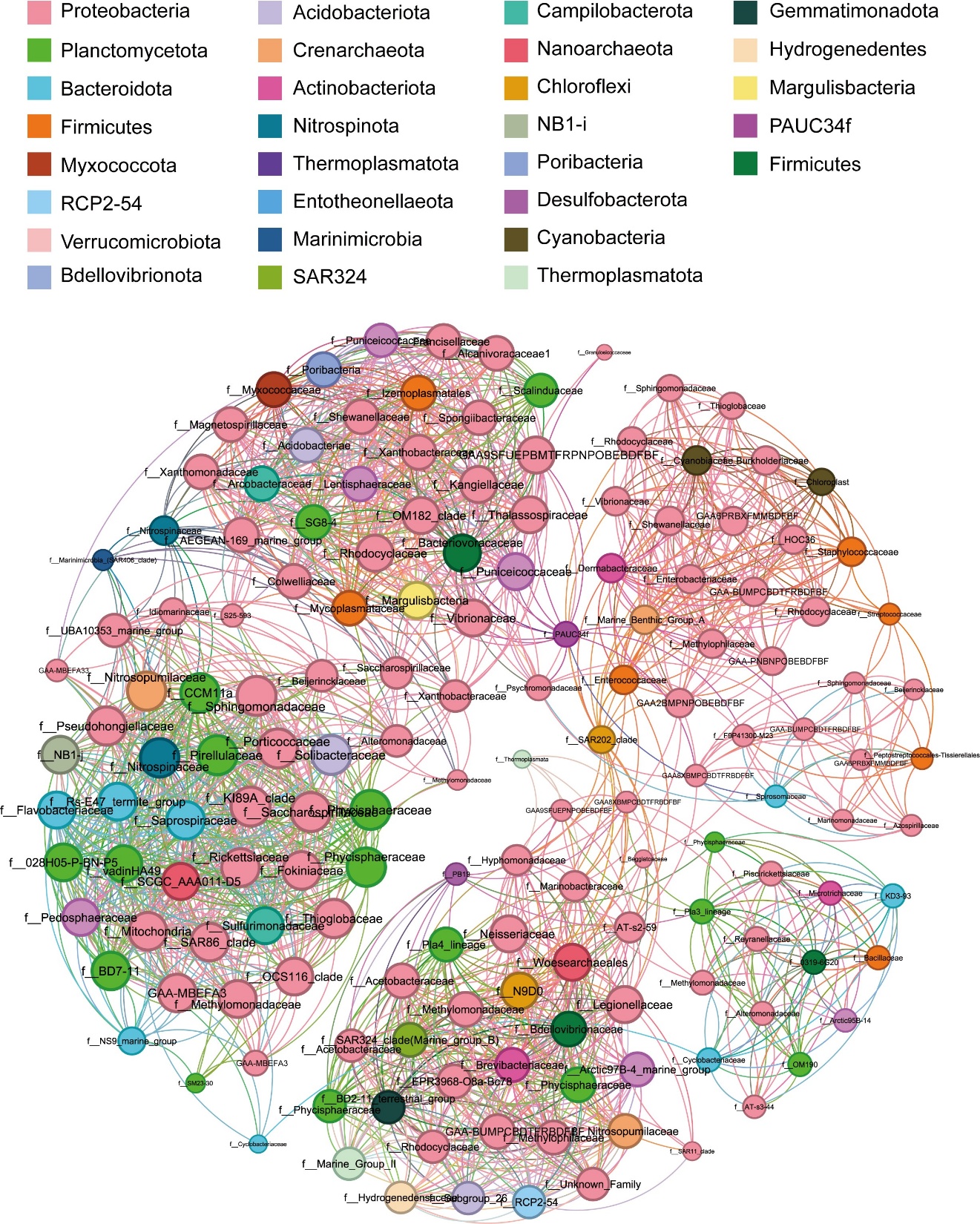


Figure S12. Co-occurrence network of prokaryotic communities based on 16S rRNA gene sequences in the metatranscriptomes for the summer samples.

The nodes in network are colored by phylum and marked by family. Only nodes (OTUs) that were significantly correlated each other (Spearman’s correlation > 0.8; after Benjamini and Hochberg FDR adjustment, *P* < 0.05) were connected (edges). The size of the nodes is proportional to the number of the connections. The edge thickness indicates the correlation values. Specific parameters of the constructed network refer to Tables S12.

# Figure 13


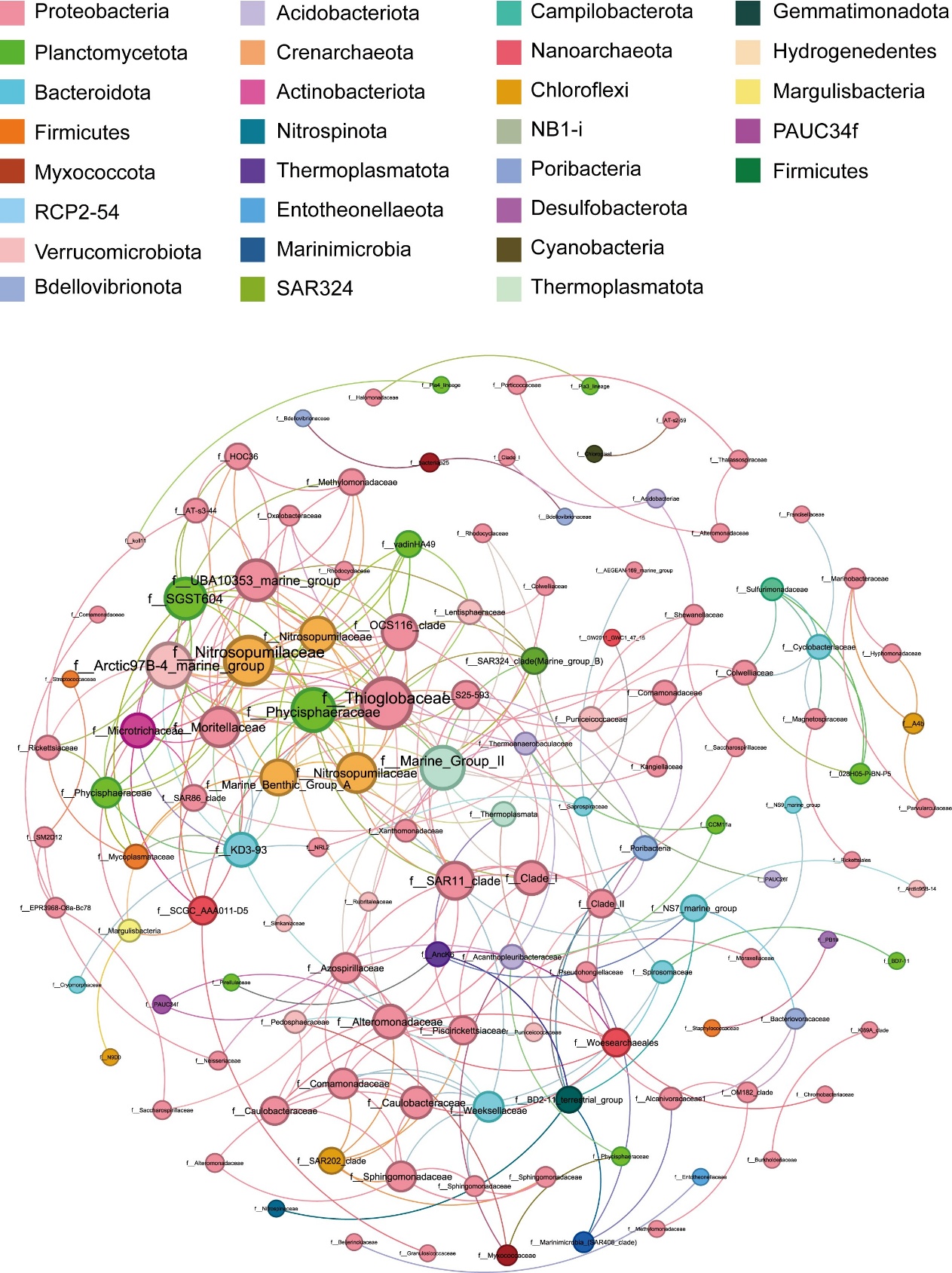


Figure S13. Co-occurrence network of prokaryotic communities based on 16S rRNA gene sequences in the metatranscriptomes for the winter samples.

The nodes in network are colored by phylum and marked by family. Only nodes (OTUs) that were significantly correlated with each other (Spearman’s correlation > 0.8; after Benjamini and Hochberg FDR adjustment, *P* < 0.05) were connected (edges). The size of the nodes is proportional to the number of the connections. The edge thickness indicates the correlation values. Specific parameters of the constructed network refer to Tables S13.

# Figure S13.


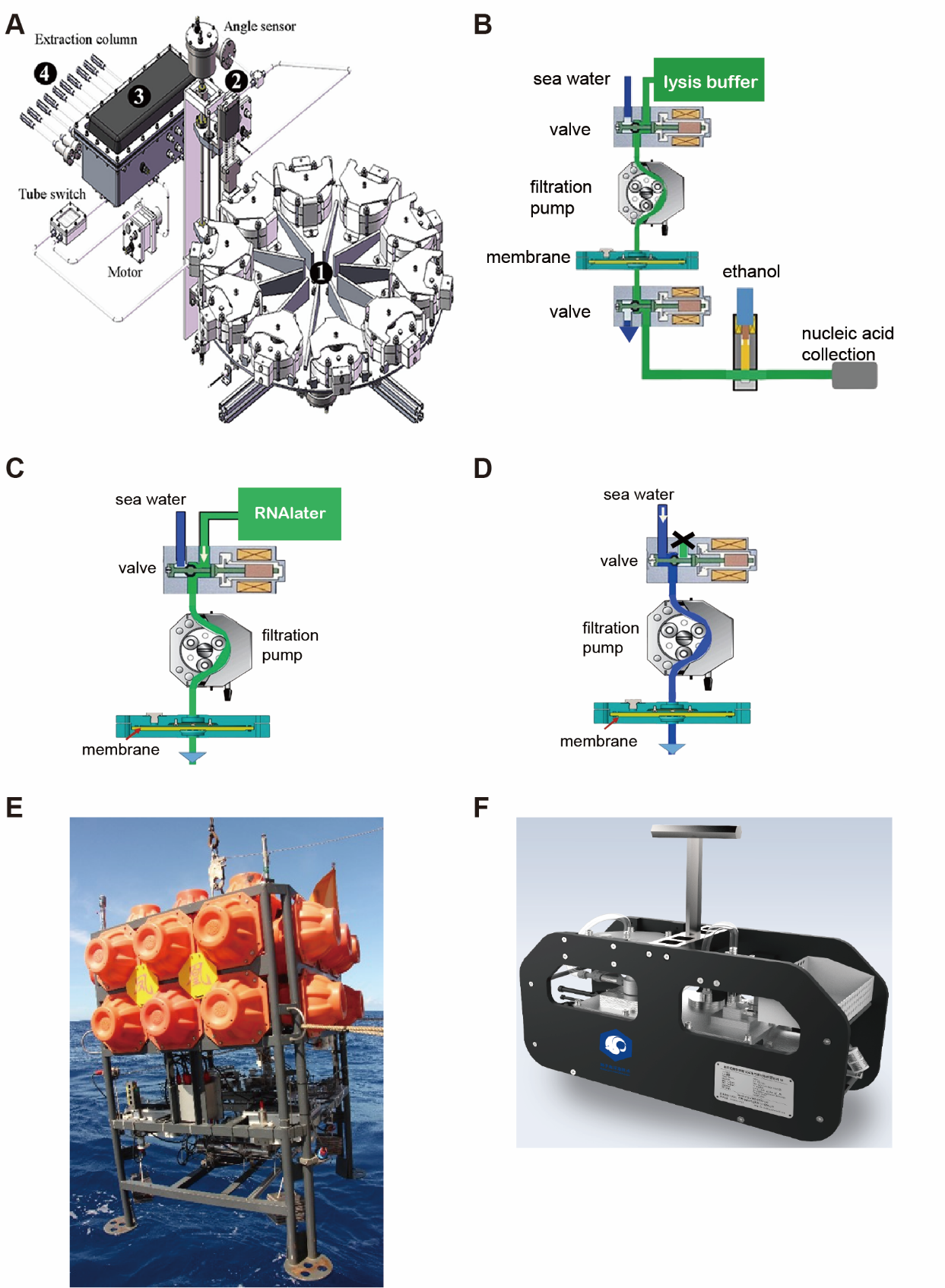


Figure S13. Schematic diagram of four deep-sea sampling methods. (A) The structural layout of the MISNAC (Multiple In Situ Nucleic Acid Collections) apparatus. The device is composed of four primary subsystems: (1) a chamber support and transition module; (2) a hose docking module; (3) a hydraulic regulation module; and (4) a nucleic acid capture module. (B) The cell lysis and DNA/RNA coextraction workflow for the six MISNAC samples following the cell filtration into the chamber of the MISNAC device. DNA/RNA was precipitated by alcohol injected into the hose and was then retrieved by a nucleic acid absorption column.
